# Polyamide-Scorpion Cyclam Lexitropsins Selectively Bind AT-Rich DNA Independently of the Nature of the Coordinated Metal

**DOI:** 10.1371/journal.pone.0017446

**Published:** 2011-05-09

**Authors:** Anthony T. S. Lo, Noeris K. Salam, David E. Hibbs, Peter J. Rutledge, Matthew H. Todd

**Affiliations:** 1 Schrödinger, Inc., New York, New York, United States of America; 2 Faculty of Pharmacy, University of Sydney, Sydney, New South Wales, Australia; 3 School of Chemistry, University of Sydney, Sydney, New South Wales, Australia; Berlin Institute of Technology, Germany

## Abstract

Cyclam was attached to 1-, 2- and 3-pyrrole lexitropsins for the first time through a synthetically facile copper-catalyzed “click” reaction. The corresponding copper and zinc complexes were synthesized and characterized. The ligand and its complexes bound AT-rich DNA selectively over GC-rich DNA, and the thermodynamic profile of the binding was evaluated by isothermal titration calorimetry. The metal, encapsulated in a scorpion azamacrocyclic complex, did not affect the binding, which was dominated by the organic tail.

## Introduction

The sequence-selective binding of small molecules to DNA is an important area of research because through such binding it may be possible to control gene expression, which has significant implications for new therapeutics.[1]–[5] Small molecule-based metal complexes are particularly sought-after in this regard since DNA binding may be used to trigger reactivity, unleashing chemical activity at a specific sequence of genetic information that is associated with disease.[6]–[7]

Many naturally-occurring small molecules are known to bind DNA with sequence selectivity, most notably the polyamide class of minor groove binders that includes distamycin and netropsin, known generically as the lexitropsins.[8]–[12] Distamycin and netropsin selectively bind AT-rich regions of DNA, sequences that are important for example because of the widespread occurrence of the TATA box transcription factor binding site in the genome.[13] Lexitropsins are structurally simple molecules possessing features that are well-suited for minor groove binding: they are curved (although this is not an absolute requirement[14]), flat and contain well-positioned hydrogen bonding groups, positively charged end groups and strategically placed van der Waals contacts.[15]–[16]

With such a well-evolved scaffold for interaction with DNA, it is unsurprising that there has been a great deal of interest in tailoring the basic design to build in greater sequence-selectivity and adapt these structures to develop new types of drugs.[17]–[36] Much has been learned about how to modify lexitropsin structures to achieve binding to bespoke DNA sequences[9], [37]–[42] or to improve physicochemical and pharmacokinetic properties.[26], [43]–[47]

There has been much interest in the attachment of chemically active groups such as alkylating agents to lexitropsins in the hope of targeting reactive chemical functionality to the double helix.[48]–[49] Given the potential of metal-based artificial nucleases and imaging agents, it is surprising that only a relatively small number of lexitropsin-metal conjugates have been reported. Dervan has described the use of a lexitropsin-EDTA-Fe complex for “affinity cleaving” near AT-rich sites.[50]–[52] Ferrocene has been used to connect two polyamide strands.[53] Iron-bleomycin analogs have been attached to lexitropsins at the *N*-[54]–[55] and *C*-[56]–[57] termini, showing that the polyamide can overturn the inherent GC-selectivity of the bleomycin portion. Bleomycin analogs have also been attached to lexitropsins in conjunction with cobalt.[58] Copper- salen,[59] -phenanthroline,[60]–[61] –peptide[62] and –bipyridine[63]–[64] complexes have been conjugated to lexitropsins, as well as a copper complex consisting of an *N*-terminal peptide and *C*-terminal intercalator.[65] Other metal complexes associated with lexitropsins include manganese,[66] vanadium,[67] tungsten,[68] platinum[69]–[71] and the radionuclide technetium-99m.[72] The first example of a zinc complex attached to a lexitropsin was only recently reported.[73] To date there have been no reports of lexitropsins bound to azamacrocycles or azamacrocyclic complexes, which is surprising given how widely such frameworks are used in coordination chemistry.[74] The thermodynamics of DNA binding with lexitropsin-metal complex conjugates have not been examined, nor has the effect of varying the metal coordinated within the same lexitropsin analog been investigated. It is also frequently the case that metal-lexitropsin conjugates are not characterized prior to their interaction with DNA, and are assumed to form *in situ*. This report addresses these areas.

We recently became interested in the attachment of azamacrocycles to motifs that recognize biological molecules. We have previously demonstrated that it is possible to influence an azamacrocycle's interaction with DNA by changing the nature of an amino acid appended to the macrocycle,[75] and created a metal complex whose primary coordination environment changes in response to the binding of a protein.[76] For a more general approach to the study of azamacrocycle-DNA interactions, a generic method for ensuring proximity of the azamacrocycle complex to DNA is required. If azamacrocycles can be reliably targeted in this way, it becomes possible to study their labeling and nuclease functions for diverse applications. This report describes the first synthesis of lexitropsin-cyclam complexes and the nature of their interaction with oligonucleotides. Cyclam was chosen as the azamacrocycle in this study since this ligand has found wide use in biology and medicine owing to its robust and well characterized coordination chemistry.[77]

## Results

### Synthesis

**Figure 1:**
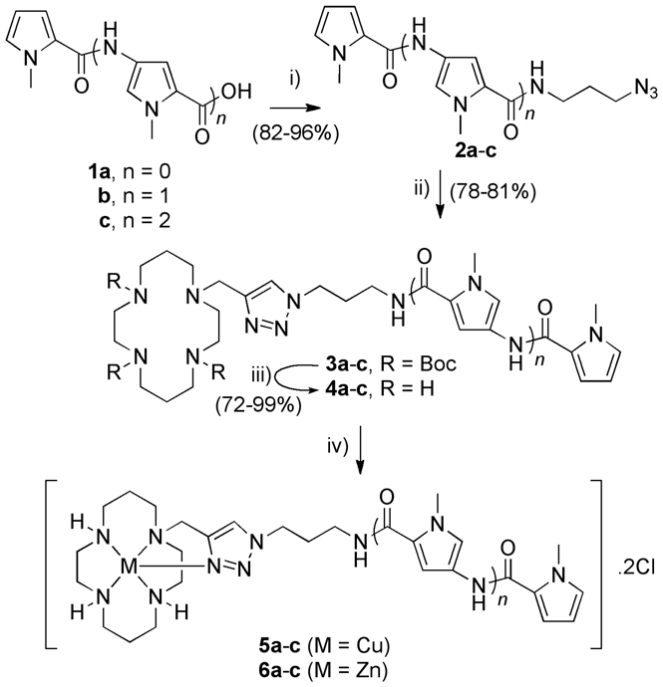
Scheme for reagents and conditions employed in ligand synthesis. i) EDC·HCl, HOBt, *N*,*N*-diisopropylethylamine, 3-azidopropylamine, dichloromethane, rt; ii) CuI (10 mol%), sodium ascorbate (20 mol%), ^t^BuOH/water (1∶1), propargyl tri-boc protected cyclam, rt; (iii) TFA/dichloromethane (1∶5), 6 h, rt, (iv) CuCl_2_ or ZnCl_2_ solution in methanol, 5 min, rt.

The targets of the synthesis were lexitropsin-cyclam conjugates **4a**–**c** (Figure 1), formed by the union of the polyamide binding motif and the azamacrocycle through the synthetically facile copper-catalyzed azide-alkyne Huisgen cycloaddition (a so-called ‘click’ reaction).[78] Compounds **1a**–**c** [79]–[80] and propargylated cyclam[81] were prepared according to literature methods (Scheme S1 and Text S1). Four aspects of these structures are of interest in comparison to literature lexitropsins: a) lack of the *N*-terminal formamido group, b) attachment of an unprecedented group (cyclam) to the C terminus, c) inclusion of an alkyl spacer between the azamacrocycle and the recognition motif and d) complexation of metal ions (copper and zinc). It was anticipated that these features would combine to provide structures capable of binding DNA, and the influence of each feature is discussed in more detail below.

#### 1) Ligand Synthesis

The pyrrole acids **1a**–**c** were coupled with commercially-available 3-aminopropyl azide to give **2a**–**c** which were coupled to the protected propargyl cyclam in good yields. Removal of the Boc groups to give the free amines proceeded smoothly. It was noted that intermediates in the synthesis of **1** containing deprotected amines (i.e. after removal of Boc groups from the aminopyrrole moiety) decomposed after a few hours at room temperature, and were therefore typically used immediately after isolation. Compounds **2** and **3** were found to be hygroscopic, but were effectively handled (and weighed) as ethereal solutions.

#### 2) Metal Complexation

**Figure 2:**
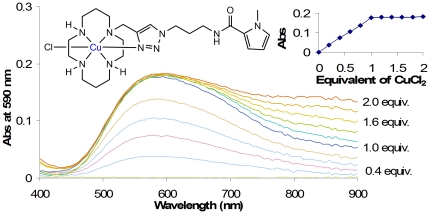
UV-vis spectrum for the titration of a solution of CuCl_2_ with compound 4a in methanol (graphical representation of raw data). The increase in absorbance reaches a maximum after the addition of 1 eq CuCl_2_ (inset).

Given the novelty of these cyclam ligands it was important to characterize their metal complexation prior to assessing their interactions with DNA. Model compound **4a**, containing a single pyrrole in the side chain, was employed for these studies as representative of the other compounds. Titration with copper(II) chloride in methanol led to the appearance of a peak in the UV-visible spectrum (λ_max_ = 590 nm, *ε* = 414 M^−1^cm^−1^) that reached a maximum absorbance with the addition of one equivalent of CuCl_2_, indicating the formation of a well-defined complex (Figure 2). The λ_max_ is similar to previously-reported scorpion cyclam complexes of copper.[76] The sharpness of the transition at one equivalent of added metal salt is notable (Figure 2, inset), and implies a high association constant between the metal ion and ligand as has been seen with related complexes (although this was not quantified as part of the current study).[81]–[82] A complexation stoichiometry of 1∶1 was confirmed by a Job plot measured at the λ_max_ of 590 nm (Figure S22; for a titration curve between CuCl_2_ and compound **4c**, see Figure S23).

**Figure 3:**
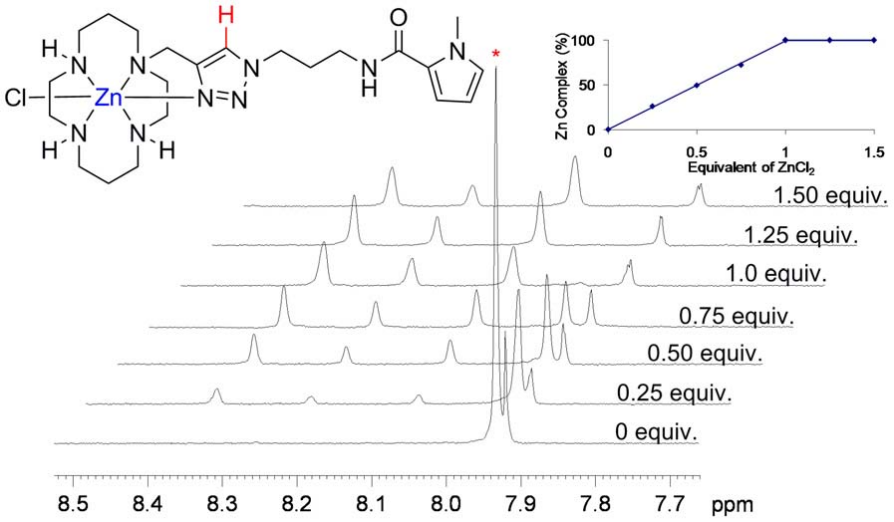
Zinc(II) chloride titration with model compound 4a monitored by ^1^H NMR spectroscopy. The ratio of the integrals of the starting material (δ 7.91 ppm) *vs*. the other peaks reaches a maximum after the addition of 1 eq ZnCl_2_ (inset).

^1^H NMR titration was used to examine the complexation between the model ligand **4a** and zinc(II) chloride in CD_3_OD by the addition of the metal salt in 0.2-equivalent increments to a solution of **4a** up to a maximum of 1.2 equivalents (Figure 3). While much of the ^1^H NMR spectrum is complex, disappearance of the signal due to the triazole proton at 7.91 ppm can be conveniently monitored during the addition. The titration clearly shows a 1∶1 complexation stoichiometry. The appearance of several new peaks in the 7.9–8.3 ppm region of the spectrum indicates the presence of interconverting species in solution that are presumably cyclam conformational isomers/diastereomers. This is supported by an approximately 1∶1 correspondence between the integral for the peaks shown at 0 equivalents of added ZnCl_2_ and the new peaks shown in the spectrum after addition of 1.50 equivalents of metal salt.

### Isothermal Titration Calorimetry

The DNA binding characteristics of cyclam-lexitropsin conjugates **4**, **5** and **6** were examined using two palindromic oligonucleotides d-(GGGATATATCCC)_2_ (oligo **I**) and d-(GGGCGGCCGCCC)_2_ (oligo **II**). The GC rich ends were chosen to stabilise the DNA duplex and encourage annealing; these sequences have melting temperatures of 36°C and 48°C respectively, meaning that they are duplexes under the conditions of the ITC experiments (25°C). The middle section of the oligonucleotide sequences was designed to probe for AT *vs*. GC selectivity, and the question arose as to whether the exact sequence of the bases in the variable region is important. Netropsin binds less well to alternating AT sequences than continuous runs (2 or more) of the two bases.[83] Bisbenzimidazole minor groove binders are very sensitive to the precise arrangement, and even sequence direction, of the bases within an AT-rich sequence[84] yet synthetic hairpin polyamides do not appear to exhibit this sensitivity.[85] Given the difficulty of predicting the behaviour of a novel lexitropsin, no attempt was made to pre-judge the behaviour of the present complexes and design specific cognate sequences. However a d(polyA).d(polyT) sequence was avoided since such oligonucleotides have unusual structures and hydration characteristics that might obfuscate a fair comparison with the GC-rich sequence.[86]–[88] The middle sequence of six bases is long enough to give meaningful binding data based on what is known of the distamycin/netropsin binding site.[15]–[16], [42], [89] and the n+1 rule of thumb of lexitropsin binding.[90] Short, model oligomers of this type are accurate models for binding characteristics with longer DNA sequences.[91]

DNA binding studies with small molecules are very sensitive to the salt concentration of the solution.[65], [79]–[80], [92] HEPES buffer was chosen for all experiments based on literature precedents.[63], [93] Ethylenediaminetetraacetic acid (EDTA) is sometimes also employed in DNA binding experiments of this type, but was not added in the present study since its metal-coordinating ability has the potential to make the role of the metal in the ligand complex ambiguous.

The concentrations of oligonucleotide and complex were 10 µM and 1000 µM respectively. Each injection (2 µL) by the calorimeter contained 1 equivalent of ligand with respect to the oligonucleotide. Control titrations were performed with ethidium bromide to validate this experimental method. EtBr was chosen for convenience; despite being an intercalator, it was important to verify correspondence between experimental and literature ITC values. The values obtained for coordination of ethidium bromide with the AT-rich oligonucleotide (ΔG = −27.6 kJ mol^−1^, ΔH = −44.8 kJ mol^−1^, ΔS = −56.9 J mol^−1^ K^−1^) are in broad agreement with those in the literature for the titration between ethidium bromide and the related poly[d(A-T)]-poly[d(A-T)] (ΔG = −38.1 kJ mol^−1^, ΔH = −41.8 kJ mol^−1^, ΔS = −12.6 J mol^−1^ K^−1^),[87] and as expected given its intercalative binding mode, similar binding constants were obtained for the AT-rich and GC-rich oligonucleotides (*ca*. 0.7×10^5^ M^−1^).

The data obtained gave K_a_, ΔH and ΔS values for each titration, as well as stoichiometry of binding; values of ΔG are calculated (Table 1). No detectable binding was observed between either oligonucleotide and the mono- or di-pyrrole compounds **4a** and **4b**, cyclam itself and its copper and zinc complexes, as well as a cyclam-triazole compound (plus its copper and zinc complexes) with a benzyl sidechain in place of the oligopyrrole moiety.[76] (See Figure S24)

**Table 1:** Binding data for selected ligands and complexes with d-(GGGATATATCCC)_2_ (**I**) and d-(GGGCGGCCGCCC)_2_ (**II**). nd = no detectable binding.

Sample	Oligo	No. sites	K_a_ (×10^5^ M^−1^)	ΔH (kJ mol^−1^)	ΔS (J mol^−1^ K^−1^)	ΔG (kJ mol^−1^)
**4c**	II	nd	-	-	-	-
	I	2.3±0.1	2.7±0.3	−19.9±0.6	37.4±2.2	−31±1.8
**5c**	II	nd	-	-	-	-
	I	2.3±0.2	1.2±0.2	−20.2±1.7	29.5±5.9	−29±4.9
**6c**	II	nd	-	-	-	-
	I	2.5±0.2	1.5±0.3	−20.8±2.0	29.5±6.9	−30±5.7

All entries are averages of two titration experiments. For a description of the calculation of errors, see Text S1.doc and Spreadsheet S1.xls.

**Figure 4:**
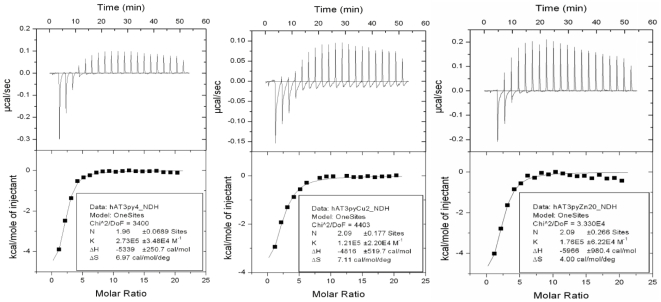
Representative binding affinity data for ligand 4c (left), 5c (centre) and 6c (right) to A•T-rich oligonucleotide I.

Strong binding was observed between the three-pyrrole conjugate **4c** and both of its metal complexes **5c** and **6c** with the AT-rich oligonucleotide **I** (Figure 4), but no binding was observed between any of these compounds and the GC-rich oligonucleotide **II**. The strength of the interactions between the AT-rich oligonucleotide and the unmetallated ligand **4c**, its copper complex **5c** and zinc complex **6c** were approximately of the same magnitude (Table 1).

## Discussion

### General remarks

The binding of the three-pyrrole compound and its complexes to AT-rich DNA occurred with a binding constant of *ca.* 1–3×10^5^ M^−1^. This strength of association compares favourably with other metal complex derivatives of lexitropsins noted in the introduction and related three-pyrrole lexitropsins,[89] but is less than that of natural lexitropsins such as distamycin itself, which has a reported K_a_ of *ca*. 3×10^8^ M^−1^ for related sequences.[87]

### Selectivity of binding

While compound **4c** and its metal complexes bind the AT-rich oligonucleotide **I** reasonably strongly, there is no detectable binding with the GC-rich oligonucleotide **II**, indicating that these lexitropsins distinguish AT-rich regions of DNA very effectively. It is usual for lexitropsins to exhibit a selectivity for certain regions of bases, but typically some binding is observed between the lexitropsin and non-cognate sequences; for example netropsin binds to poly[d(GC)].poly[d(GC)] with 38% of the enthalpy change with which it binds poly[d(AT)].poly[d(AT)].[91] The complete absence of observable binding with the GC-rich sequence, as is the case here, is unusual. This level of selectivity presumably arises from multiple disfavoured interactions in the binding with the GC-rich oligonucleotide; the enthalpic penalty for base:lexitropsin mismatch is not linearly additive, with single mismatches being quite well tolerated far better than multiple mismatches.[94]

### Number of pyrroles required for binding

The results above clearly show that three pyrroles are required for synthetic lexitropsins of this type to bind to AT-rich DNA, a figure that is consistent with the literature for related compounds.[1], [79] While naturally-occurring netropsin has only two pyrroles, the two charged groups at either end of the structure (and analogs[58], [68]) can compensate by giving rise to favourable electrostatic interactions with the helix.[16]

### Cyclam as a new C terminal modification

The greatest variation in the structure of these new lexitropsins compared to known analogs is the addition of cyclam (an alkylamine ring) to the C-terminus. A C-terminal methylene spacer between the pyrrole rings and the cyclam was employed in the design, since methylene groups form favourable van der Waals interactions with terminal A/T base pairs,[95] and the attachment of alkylamines to lexitropsins without such a spacer leads to poor DNA binding characteristics.[96]

Cyclam is an important modification because the nature of the C-terminal alkylamine can significantly alter lexitropsin binding strength. Apparently trivial changes to the alkylamine tail of lexitropsins can change their binding affinity for their cognate sequence by up to two orders of magnitude (Table S1, Entries 1–2).[96] Significant changes in the identity of the heterocyclic bases in lexitropsins with alkylamine tails can affect their binding abilities to a lesser degree (Table S1, Entries 3–4).[42] Thus while selectivity for nucleic acid sequences can obviously be imparted by certain sequences of Py and Im components, the nature of the alkylamine tail also makes an essential contribution to the overall binding strength.

The lexitropsin conjugates described herein clearly show that cyclam is well tolerated as a C-terminal modification to natural minor groove binders. Both the unmetallated ligand and metal complexes containing zinc and copper are tolerated to approximately the same degree, though the former has a slightly higher binding affinity. While this may at first seem surprising on purely electrostatic grounds (discussed further below), it should be remembered that the unmetallated cyclam ring, drawn as neutral in Figure 1 will be doubly protonated at neutral pH.[97]

Interestingly C-terminal alkylamine tails on other minor groove binders can act as a GC-directing motif, for example the piperazine ring in the compound Hoechst 33258, which exerts this change essentially on the steric grounds of requiring a wider minor groove.[98] The azamacrocycle cyclam does not have this effect in analogs **4c**–**6c**.

### N-Terminal changes

Removal of the *N*-formamido moiety from lexitropsins can significantly reduce their binding affinity for DNA,[89], [99] but does not necessarily eliminate it.[100] Many analogs are known in which this group has been replaced with related structures that modify binding affinities,[44], [99], [101]–[102] and significant changes in this region have been tolerated, for example some of the metal complex-lexitropsin conjugates described in the introduction.[55], [60], [65], [72] However, the reduction in binding affinity for Py-Py-Py (the lexitropsin scaffold of interest here) when the *N*-formamido moiety is removed is smaller than for other lexitropsins (one order of magnitude, from *ca*. 10^5^ to *ca*. 10^4^ M^−1^ for formamide-PyPyPy *vs*. PyPyPy, Table S1, Entries 5–6).[89] It is thought that the formamide affects the way the molecule stacks as a dimer in the minor groove,[89] but poly-Py lexitropsins can bind as monomers.[42] The effect of removing the *N*-formyl group also varies with lexitropsin structure, and the effects are different for hairpin- and cross-linked lexitropsins.[103] As might be expected from these observations, the binding affinities observed for the novel lexitropsin conjugates in the present work imply that the removal of the terminal *N*-formamido is not prohibitive for binding.

### Metal preference and metallated vs. unmetalated ligands

The cyclam-lexitropsin conjugates described here show essentially the same binding characteristics whether the cyclam is unmetalated *vs*. when copper or zinc is coordinated. The implication is that the metal complex plays no role in binding. The similar size of these conjugates to literature examples in which the metal is known to interact with the DNA, suggest that the cyclam should be geometrically able to do so. One possible explanation for the apparent absence of metal-DNA interactions in our systems is that the scorpion ligand structure, in which the triazole is coordinated to the metal ion, effectively hides the metal and prevents it from binding the oligonucleotide. In contrast to previous results with an avidin/biotin couple,[76] it appears that binding of the DNA does not lead to altered metal coordination in the scorpion complex. In a report of a cobalt-bleomycin-lexitropsin compound the metal-free ligand had a binding affinity with its target (4.75×10^4^ M^−1^) that was only slightly lower than that for the metalated version (2.26×10^5^ M^−1^) and a similar “shielding” of the metal from the DNA backbone by bulky ligand substituents was proposed.[58] In contrast Li *et al.* recently reported a Zn-lexitropsin conjugate based on the *bis*(2-benzimidazolyl-methyl)amine scaffold, in which the metal is available for coordination, and which exhibited a 3-fold enhancement of affinity for AT-rich oligonucleotides compared to the metal-free ligand.[73]

**Figure 5:**
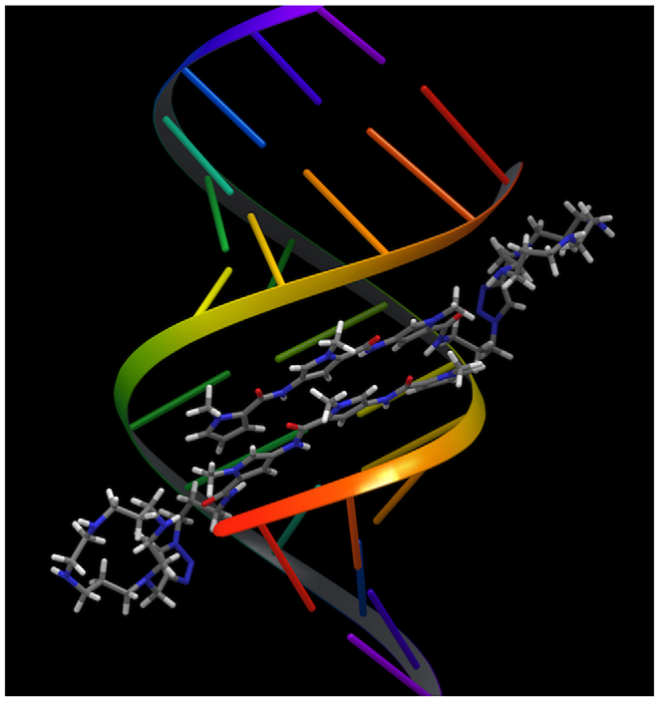
Model of the interaction between the AT-rich oligonucleotide and ligand 4c.

To verify whether the cyclam in the ligand is well placed to form favourable interactions with the phosphate backbone, molecular modeling was carried out on the complex formed between the AT-rich oligonucleotide and compound **4c** (as representative of all the ligands tested). The interaction was modeled by taking the geometry-optimized DNA oligonucleotide and after inserting an optimized dimer of cyclam ligands into the minor groove, the resulting DNA-dimer complex was then subjected to geometry optimization. The results of this procedure can be seen in Figure 5. Whether a lexitropsin tail is in the correct position to interact with the minor groove depends on both the lexitropsin structure and its mode of binding.[104] It is clear here, however, that the expected binding mode is observed for the lexitropsin in the minor groove (offset stacked dimer), yet the cyclam is situated well outside the double helix and appears to form no favourable interactions with the DNA backbone. An identical mode of binding was seen when one of the metal complexes (**6c**) was modeled in this way.

### Enthalpic vs. entropic components of binding

A consideration of both binding enthalpy and entropy is important, rather than solely the binding free energy, since enthalpic and entropic changes in small molecule-DNA binding can compensate for one another to give a misleading free energy change.[87] ITC can give valuable information above and beyond what may be gleaned from other analytical methods.[105]–[106] Certain mechanisms of DNA binding can give rise to specific signatures in the resulting thermodynamic data – thus minor groove binding interactions tend to entropically driven, while intercalation is often enthalpically-driven; lexitropsins are an exception to this rule of thumb and the –TΔS term for lexitropsin-DNA binding can be large.[107]

The lexitropsins **4c**–**6c** do not show the enthalpy-entropy compensation that is expected[87], [108] but not absolutely required[109] in drug-receptor interactions. The binding is enthalpy-dominated, but not overwhelmingly so, with entropy accounting for 30–40% of the change in free energy upon binding. The entropic gain is largest for the unmetallated ligand. The favourable gain in entropy upon binding the lexitropsins may arise from the loss of some DNA-bound water from the ‘spine of hydration’.[110]–[111] though there is still disagreement as to whether there is net water loss or gain upon minor groove binding more generally.[107] The fact that, in contrast to distamycin itself,[87] this gain in entropy is not offset by the sizeable conformational constraint imposed on the lexitropsin by the binding event,[16], [112] may be due to the lower binding affinity of these synthetic *vs*. the natural ligands.

### Binding stoichiometry

The compounds in the present work bind with a 2∶1 stoichiometry to AT-rich oligonucleotides, despite being pyrrole rich and being potentially multiply charged under the conditions employed. It is known that lexitropsins can bind to DNA and oligonucleotides with either a 1∶1[16], [113] or 2∶1[114]–[116] stoichiometry depending on factors including the nucleobase sequence and the identity and concentration of the ligand.[117] The level of cooperativity in binding also depends on the base sequence and the nature of the polyamide.[42], [118] Pyrrole-based polyamides (in contrast to those containing other heterocycles such as imidazoles) often bind with negative cooperativity, which can arise from a positive enthalpic cooperativity but strongly unfavourable entropic factors for the binding of the second ligand.[119]–[120] However, there are cases where little cooperativity is shown.[121] It is sometimes expected that pyrrole-based lexitropsins will bind with 1∶1 stoichiometry because DNA sequences consisting exclusively of A and T bases have a narrower minor groove, but this is not always the case.[115] Charge is an important factor in determining binding stoichiometry; it is expected that monocationic lexitropsins will bind oligonucleotides with a 2∶1 stoichiometry, unlike dicationic netropsin that typically binds with 1∶1 stoichiometry.[119]

Given the 2∶1 binding stoichiometry of lexotropsins **4c**–**6c** to oligo **I**, it might be expected that the association constants for the first and second binding events could be deconvoluted, or that the two binding events would be clear from a discontinuity in the ITC data. However since there is no such discontinuity, binding is either statistical (no cooperativity) or there is cooperativity but two molecules of the lexitropsin bind simultaneously to a single oligonucleotide, rather than in a statistical 1∶1 binding.[118], [120] Such cooperativity has been shown for the binding of distamycin to d(CGCATATATGCG)_2_.[122] Hence the value for K_a_ should formally be thought of as a combination of the two contributing binding events, i.e. (K_1_K_2_)^1/2^.

### Conclusion

The magnitude and selectivity of the binding exhibited by these cyclam-polyamide compounds is gratifying for the reasons detailed above. Despite lacking a terminal formamide, not necessarily incorporating an optimized DNA sequence for binding, and in the face of literature precedent showing that unoptimised alkylamines can significantly reduce the binding efficiency of lexitropsins, the K_a_ values observed for the three conjugates that exhibit binding are high, with complete selectivity for the AT-rich oligonucleotide over the GC-rich sequence. The data (and modeling) show that in the cases studied, there was little influence of the nature of the cyclam and coordinated metal on the degree of DNA binding. This arises because once the lexitropsin binds as a dimer in the minor groove, the cyclam is positioned beyond the backbone of the DNA helix.

There is considerable scope for modifying these structures to optimize binding, and to position the cyclam and its complexes for interaction with the DNA backbone. Of particular interest will be to vary the structure of the scorpion ligand to facilitate metal interaction with the DNA helix upon binding, so as to permit the future development of sequence-specific DNA cleavage. Future study of the potential nuclease activity of the metal centre would likely employ the related azamacrocycle cyclen, the metal complexes of which are known to promote phosphodiester cleavage in model systems[123] and AT-specific oligonucleotide binding (when conjugated to intercalating moieties).[124] The synthetic accessibility of these conjugates makes such optimization and diversification straightforward.

Another future application of complexes of this type is as imaging agents for the presence of specific DNA sequences using complexes whose optical properties change upon binding. The attachment of cyclams also offers potential improvements in the cell permeability of the resulting lexitropsins: it is known that zinc sensors based on related triazole-cyclam motifs are cell-permeable,[81] while hairpin polyamides themselves have limited cellular penetration.[9]

## Materials and Methods

### A) General Procedures

#### Synthesis

Novel compounds are described below; all other compounds are described in the Scheme S1 and Text S1. The procedure used for the couplings of the 1-methylpyrroles into longer chains was adapted from literature[79]–[80] but using EDC·HCl and HOBt as the coupling reagents. The oligonucleotides d-(GGGATATATCCC)_2_ and d-(GGGCGGCCGCCC)_2_ and were purchased from Geneworks (Adelaide, Australia; HPLC purified). Reagents were obtained from Sigma Aldrich, Fluka, Novabiochem or Alfa Aesar and used directly without further purification. Milli-Q water was used in all physical measurements. NMR spectra for novel compounds are provided along with the .dx files (NMR Data S1.zip) which may be read by any NMR processing software.

#### UV-vis

UV-vis spectra were recorded on a Cary 4E UV-vis spectrophotometer between 290 and 900 nm using a 1 cm×1 cm quartz cuvette. For the copper(II) complex titration experiment, measurements were taken of cyclam complex (1.0 eq) dissolved in methanol (1 mL). Copper(II) chloride (73.4 mM) was added in 0.2 eq aliquots until 2 eq had been added. Measurements were taken after 30 s of stirring. For the Job plot a series of metal and ligand mixtures was prepared, such that the total molarity was the same while changing the metal and ligand ratio at 0.2 eq intervals.[125] The maximum absorbance obtained from these solutions at a particular wavelength was plotted against the mole ratio of ligand.

#### NMR

^1^H and ^13^C Nuclear Magnetic Resonance spectroscopy was performed on either a Bruker Avance DPX 200 Spectrometer or a Bruker Avance DPX 300 Spectrometer. For the zinc titration experiment, the cyclam ligands were dissolved in CD_3_OD (to 5.6 mM) and a solution of zinc(II) chloride in CD_3_OD (73.4 mM) was titrated to 1.2 eq in 0.2 eq increments.

#### Calorimetry

DNA binding studies were performed on an iTC200 Microcalorimeter made of Hastelloy® Alloy C-276. The system was operated at 25°C with a coin cell design with a capacity of 200 µL and a titration syringe with a capacity of 40 µL. The amount injected was 2 µL per 150 seconds with a stirring rate of 1000 rpm. The stock solution of DNA in the calorimeter chamber was 10 µM in 10 mM HEPES buffer containing 100 mM NaCl and the ligand. The stock ligand solution (1000 µM) was diluted to a concentration of 10 µM with the buffer solution prior to ITC experiments and was titrated into the DNA solution. Single stranded DNA oligos were supplied by Geneworks and dissolved in buffer (10 mM HEPES, 100 mM sodium chloride, pH 7.0) and shaken gently at 25°C for 2 days to yield double stranded oligonucleotides to a stock concentration of 100 µM determined using a Nanodrop 1000 spectrophotometer (Thermo scientific version 3.6.0). A correction was made for the heat of dilution of the ligands, estimated from the peaks obtained from injections at the end of a given ITC experiment (following saturation).

Metal complex synthesis for ITC experiments: to the ligand (1 eq) was added a solution of copper(II) chloride solution (73.4 mM, 1.0 eq) in methanol or zinc(II) chloride solution (73.4 mM, 1.0 eq) in methanol. The methanol was removed under reduced pressure and HEPES buffer (10 mM with 100 mM NaCl) was added to obtain a final stock ligand concentration of 1000 µM which was kept at 0°C. These complexes were used directly in DNA binding studies.

### B) Typical General Synthetic Procedures

#### Peptide coupling (A)

To the carboxylic acid (1.0 eq) and amine (1.3 eq) in anhydrous dichloromethane (solution is *ca*. 125 mM in acid) were added EDC·HCl (1.2 eq), HOB*t* (1.2 eq) and *N*,*N*-diisopropylethylamine (3.0 eq). The reaction mixture was stirred at rt under nitrogen for 12 h. Sodium bicarbonate solution (10% w/v) was added dropwise to the reaction mixture until pH 10 was reached and the reaction mixture was extracted with dichloromethane (3 times). The combined organic phases were dried (Na_2_SO_4_) and concentrated under reduced pressure.

#### ‘Click’ reaction (B)

Alkyne (0.93 eq) and azide (1.0 eq) were dissolved in a mixture of water/tert-butanol (1∶1, to give 100 mM solution in azide) and stirred at 27°C under nitrogen. A solution of copper(II) sulfate pentahydrate (0.31 mmol, 0.1 eq) and sodium ascorbate (0.62 mmol, 0.2 eq) in water (to give a solution that was 125 mM in copper) was added to the reaction mixture and stirring was continued for 16 h. The reaction was quenched with saturated sodium bicarbonate solution until pH 10 was reached and the mixture extracted with dichloromethane (3 times). The combined organic phases were dried (Na_2_SO_4_) and concentrated under reduced pressure.

#### TFA deprotection of Boc groups (C)

To the Boc-protected compound (1.0 eq) in anhydrous dichloromethane (300 mM) was added trifluoroacetic acid (10 eq) dropwise and stirring was continued at rt for 6 h. The reaction was cooled to 0°C before the addition of water (same volume as dichloromethane). Sodium hydroxide (1 M) was added dropwise until pH 10 was reached. The mixture was extracted with chloroform (3 times). The combined organic phases were dried (Na_2_SO_4_) and concentrated under reduced pressure.

#### Base deprotection of ester group (D)

To the ester-protected compound (1.0 eq) in a mixture of water/methanol (1∶1, 5 mM) was added sodium hydroxide (0.25 M, 4.0 eq) and the solution was heated at reflux for 3 h under nitrogen. The reaction mixture was washed with ethyl acetate (2 times) and the aqueous phase was acidified to pH 3 with hydrochloric acid (1 M) and was extracted with ethyl acetate (3 times). The combined organic phases were dried (Na_2_SO_4_) and concentrated under reduced pressure.

#### Complexation of copper(II) and zinc(II) cyclam derivatives (E)

To *N*-functionalized cyclam (1.0 eq) was added copper(II) chloride or zinc(II) chloride solution in methanol (73.4 mM, 1.0 eq) and stirring was continued at rt for 10 min. Methanol was evaporated *in vacuo* and HEPES buffer (10 mM containing 100 mM NaCl) was added to give a final ligand concentration of 1000 µM.

### C) Molecular Modeling

DNA oligonucleotide d-(GGGATATATCCC)2 was constructed as the B-form regular helix using the Maestro 9.1 (Maestro, v9.1.107, Schrödinger, LLC) graphical user interface. Cyclam ligand structures were built, manipulated and adjusted for chemical correctness using Maestro, employing MacroModel 9.8 (Macro-Model, v9.8, Schrödinger, LLC). Geometry minimizations were performed on all cyclam ligands using the OPLS_2005 (MacroModel) force field and the Truncated Newton Conjugate Gradient (TNCG). Optimizations were converged to a gradient RMSD below 0.05 kJ/mol or continued to a maximum of 1000 iterations, at which point there were negligible changes in RMSD gradients.

### D) Synthesis of Novel Compounds

#### *N*-(3-Azidopropyl)-1-methylpyrrole-2-carboxamide 2a

1-Methylpyrrole-2-carboxylic acid **1a** (0.24 g, 2.0 mmol, 1 eq) and 3-azidopropylamine (0.26 g, 2.6 mmol, 1.3 eq) were coupled using general procedure A with purification by flash column chromatography (1∶1 ethyl acetate/hexane, *R*_F_ 0.31) yielding **2a** (0.34 g, 82%) as a light yellow oil; **IR** (ATR) 2091, 1631 cm^−1^; **^1^H NMR** (200 MHz, CDCl_3_) δ 6.69–7.72 (1H, m, Ar), 6.51 (1H, dd, *J* 3.9 & 1.6, Ar), 6.07 (1H, dd, *J* 3.9 & 2.6, Ar), 5.96–6.05 (1H, br s, NH), 3.94 (3H, s, CH_3_), 3.46 (2H, t, *J* 6.6 Hz, H^1^), 3.41 (2H, t, *J* 6.6 Hz, H^3^), 1.86 (2H, qn, *J* 6.6 Hz, H^2^) (Figure S1); **^13^C NMR** (50.3 MHz, CDCl_3_) δ 161.9 (C = O), 127.4 (Ar), 125.2 (Ar), 111.5 (Ar), 106.7 (Ar), 48.8, 36.2, 36.0, 28.6 (Figure S2); **MS** (APCI) m/z 108.0 (C_6_H_8_NO^+^, 86%), 208.0 (MH^+^, 29%); **HRMS** (APCI) calcd for C_9_H_14_N_5_O^+^ 208.11984 found 208.11929 (MH^+^).

#### *N*-(3-Azidopropyl)-1-methyl-4-(1-methyl-1*H*-pyrrole-2-carboxamido)-1*H*-pyrrole-2-carboxamide 2b

Methylpyrrole amide carboxylic acid **1b** (104 mg, 0.42 mmol, 1 eq) and 3-azidopropylamine (55 mg, 0.55 mmol, 1.3 eq) were coupled according to procedure A, with purification by flash column chromatography (ethyl acetate, *R*_F_ 0.59) yielding **2b** (122 mg, 88%) as a light yellow oil; **IR** (CHCl_3_) 3326, 2096, 1640, 1535 cm^−1^; **^1^H NMR** (300 MHz, CDCl_3_) δ 7.74 (1H, br s, NH), 7.09–7.11 (1H, m, Ar), 6.73–6.77 (1H, m, Ar), 6.66–6.70 (1H, m, Ar), 6.55–6.57 (1H, m, Ar), 6.05–6.15 (2H, m, Ar), 3.95 (3H, s, NCH_3_), 3.86 (3H, s, NCH_3_), 3.35–3.55 (4H, m, C*H*_2_CH_2_C*H*_2_N_3_), 2.74–3.25 (1H, br s, NH), 1.77–1.88 (2H, m, CH_2_C*H*_2_CH_2_N_3_) (Figure S3); **^13^C NMR** (75.5 MHz, CDCl_3_) δ 161.8 (C = O), 159.4 (C = O), 128.5 (Ar), 125.4 (Ar), 123.2 (Ar), 121.3 (Ar), 118.9 (Ar), 112.0 (Ar), 107.4 (Ar), 103.5 (Ar), 49.4, 36.9, 36.8, 36.5, 28.9 (Figure S4); **HRMS** (APCI) calcd for C_15_H_19_N_7_NaO_2_^+^ 352.14979 found 352.14924 (MNa^+^).

#### *N*-(3-Azidopropyl)-1-methyl-4-(1-methyl-4-(1-methyl-1*H*-pyrrole-2-carboxamido)-1*H*-pyrrole-2-carboxamido)-1*H*-pyrrole-2-carboxamide 2c

Pyrrole amide carboxylic acid **1c** (104 mg, 0.42 mmol, 1 eq) and 3-azidopropylamine (55 mg, 0.55 mmol, 1.3 eq) were coupled using procedure A. The residue was purified by flash column chromatography (ethyl acetate, *R*_F_ 0.59) yielding **2c** (122 mg, 96%) as a light yellow oil; **^1^H NMR** (300 MHz, CDCl_3_) δ 8.46 (1H, br s, NH), 8.15 (1H, br s NH), 8.05 (1H, br s, NH), 6.88–6.94 (3H, m, Ar), 6.80–6.82 (1H, m, Ar), 6.68–6.71 (1H, m, Ar), 6.61–6.67 (1H, m, Ar), 6.21–6.25 (1H, m, Ar), 4.09 (3H, s, NCH_3_), 3.98 (3H, s, NCH_3_), 3.95 (3H, s, NCH_3_), 3.42–3.70 (4H, m, C*H*_2_CH_2_C*H*_2_N_3_), 1.93–2.10 (2H, m, CH_2_C*H*_2_CH_2_N_3_) (Figure S5); **^13^C NMR** (75.5 MHz, CDCl_3_) δ 161.9 (C = O), 159.6 (C = O), 159.0 (C = O), 128.4 (Ar), 125.3 (Ar), 123.2 (Ar), 122.9 (Ar), 121.5 (Ar), 121.3 (Ar), 119.5 (Ar), 119.0 (Ar), 112.3 (Ar), 107.3 (Ar), 104.0 (Ar), 103.5 (Ar), 49.2, 36.8, 36.7, 36.4, 36.4, 28.8 (Figure S6); **MS (**ESI**)** m/z 452.1 (MH^+^, 68%), 474.3 (MNa^+^, 75%); **HRMS** (ESI) calcd for C_21_H_25_N_9_NaO_3_^+^ 474.19781 found 474.19726 (MNa^+^).

**Figure 6:**
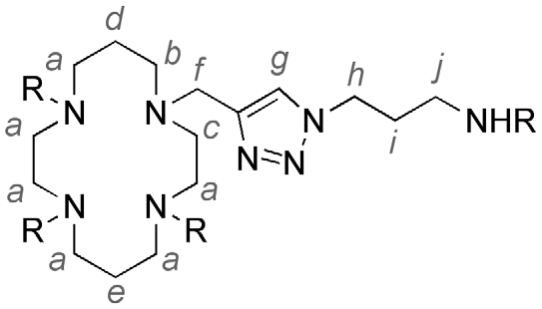
NMR spectroscopy assignment convention for molecules containing cyclam.

For cyclam-based compounds an NMR assignment convention is used as shown in Figure 6.

#### Tri-*tert*-butyl 11-((1-(3-(1-methyl-1*H*-pyrrole-2-carboxamido)propyl)-1*H*-1,2,3-triazol-4-yl)methyl)-1,4,8,11-tetraazacyclotetradecane-1,4,8-tricarboxylate 3a[76]

Tri-Boc propargyl cyclam (0.70 g, 1.30 mmol, 0.93 eq) and azide **2a** (0.29 g, 1.4 mmol, 1 eq) were reacted together according to procedure B, giving a white gum which was purified by flash column chromatography (ethyl acetate, *R*_F_ 0.20) yielding **3a** (0.79 g, 81%) as a white solid; **mp** 72–74°C; **IR** (ATR) 3366, 1687, 1544 cm^−1^; **^1^H NMR** (300 MHz, CDCl_3_) δ 7.51 (1H, br s, H^g^), 6.69–6.72 (1H, m, Ar), 6.59–6.66 (1H, m, Ar), 6.30–6.57 (1H, br s, NH), 6.05–6.09 (1H, m, Ar), 4.44 (2H, t, *J* 6.7 Hz, H^h^), 3.93 (3H, s, NCH_3_), 3.74 (2H, s, H^f^), 3.68–3.85 (2H, m, H^j^), 3.20–3.50 (12H, m, H^a^), 2.58–2.70 (2H, m, H^b^), 2.40–2.50 (2H, m, H^c^), 2.16–2.36 (2H, m, H^i^), 1.80–2.00 (2H, m, H^d^), 1.65–1.80 (2H, m, H^e^), 1.44 (27H, s, C(CH_3_)_3_) (Figure S7); **^13^C NMR** (50.3 MHz, CDCl_3_) δ 162.0, 155.4, 155.1, 127.5, 125.1, 122.6, 111.8, 79.1, 77.6, 77.0, 76.4, 59.9, 46.0–47.5 (multiple peaks), 36.3, 35.9, 30.1, 28.1 (Figure S8); **MS (ESI)** m/z 746.3 (MH^+^, 61%), 768.3 (MNa^+^, 100%), **HRMS (ESI)** calcd for C_37_H_64_N_9_O_7_^+^ 746.49287 found 746.49162 (MH^+^).

#### Tri-*tert*-butyl 11-((1-(3-(1-methyl-4-(1-methyl-1*H*-pyrrole-2-carboxamido)-1*H*-pyrrole-2-carboxamido)propyl)-1*H*-1,2,3-triazol-4-yl)methyl)-1,4,8,11-tetraazacyclotetradecane-1,4,8-tricarboxylate 3b[76]

Tri-Boc propargyl cyclam (209 mg, 0.39 mmol, 0.93 eq) and azide **2b** (138 mg, 0.42 mmol, 1 eq) were reacted together according to general procedure B yielding a light yellow oil, which was purified by flash column chromatography (ethyl acetate, *R*_F_ 0.18) yielding **3b** (284 mg, 78%) as a hygroscopic white gum; **IR** (ATR) 3337, 2974, 1681 cm^−1^; **^1^H NMR** (300 MHz, CDCl_3_) δ 8.05 (1H, s, NH), 7.50 (1H, s, H^g^), 6.55–6.80 (5H, m, Ar, N*H*), 6.08 (1H, dd, *J* 3.9, 2.6 Hz, Ar), 4.38–4.50 (2H, m, H^h^), 3.95 (3H, s, N(CH_3_)), 3.88 (3H, s, N(CH_3_)), 3.68–3.78 (2H, m, H^j^), 3.20–3.45 (14H, m, H^a,f^), 2.57–2.65 (2H, m, H^b^), 2.36–2.52 (2H, m, H^c^), 2.12–2.23 (2H, m, H^i^), 1.80–1.96 (2H, m, H^d^), 1.63–1.79 (2H, m, H^e^), 1.43 (27H, s, 3 C(CH_3_)_3_) (Figure S9); **^13^C NMR** (50.3 MHz, CDCl_3_) δ 162.1, 159.3, 155.8, 155.6, 128.3, 125.5, 122.9, 121.6, 119.1, 111.9, 107.3, 103.6, 79.6, 46.0–47.8 (several peaks), 36.8, 36.5, 36.3, 29.9, 28.5 (Figure S10); **MS (ESI)** m/z 868.4 (MH^+^, 56%), 890.6 (MNa^+^, 100%); **HRMS (ESI)** calcd for C_43_H_70_N_11_O_8_^+^ 868.54088 found 868.54034 (MH^+^).

#### Tri-*tert*-butyl 11-((1-(3-(1-methyl-4-(1-methyl-4-(1-methyl-1*H*-pyrrole-2-carboxamido)-1*H*-pyrrole-2-carboxamido)-1*H*-pyrrole-2-carboxamido)propyl)-1*H*-1,2,3-triazol-4-yl)methyl)-1,4,8,11-tetraazacyclotetradecane-1,4,8-tricarboxylate 3c[81]

Tri-Boc propargyl cyclam (47 mg, 0.088 mmol, 0.93 eq) and methylpyrrole azide **2c** (43 mg, 0.095 mmol, 1 eq) were reacted together according to procedure B yielding a light yellow oil, which was purified by flash column chromatography (ethyl acetate, *R*_F_ 0.31) yielding **3c** (73 mg, 78%) as a hygroscopic white gum; **IR** (ATR) 3316, 2975, 2935, 1685 cm^−1^; **^1^H NMR** (300 MHz, CDCl_3_) δ 8.07–8.55 (1H, br s, NH), 8.25–8.56 (2H, m, NH), 7.52 (1H, s, H^g^), 6.70–6.90 (5H, m, Ar), 6.46–6.54 (1H, m, Ar), 6.02–6.08 (1H, m, Ar), 4.39 (2H, m, H^h^), 3.92 (3H, s, NCH_3_), 3.86 (3H, s, NCH_3_), 3.82 (3H, s, NCH_3_), 3.62–3.80 (2H, m, H^j^), 3.00–3.60 (14H, m, H^a,f^), 2.52–2.65 (2H, m, H^b^), 2.30–2.50 (2H, m, H^c^), 2.10–2.22 (2H, m, H^i^), 1.80–1.95 (2H, m, H^d^), 1.60–1.75 (2H, m, H^e^), 1.42 (27H, s, 3 C(CH_3_)_3_) (Figure S11); **^13^C NMR** (50.3 MHz, CDCl_3_) δ 162.1, 159.5, 159.0, 155.8, 128.3, 125.5, 123.0, 122.8, 121.7, 119.4, 119.2, 112.2, 107.3, 104.0, 103.6, 79.6, 44–50 (several peaks), 36.7, 36.6, 30.0, 28.4 (Figure S12); **MS** (ESI) m/z 1012.8 (MNa^+^, 100%); **HRMS** (ESI) calcd for C_49_H_76_N_13_O_9_^+^ 990.58890 found 990.58835 (MH^+^).

#### *N*-(3-(4-((1,4,8,11-Tetraazacyclotetradecan-1-yl)methyl)-1*H*-1,2,3-triazol-1-yl)propyl)-1-methyl-1*H*-pyrrole-2-carboxamide 4a[81]

Monomethylpyrrole tri-Boc-protected cyclam **3a** (0.55 g, 0.74 mmol 1 eq) was deprotected according to general procedure C yielding **4a** as a colourless oil. The product was purified by reverse phase HPLC (2% CH_3_CN for 5 min, ramping to 60% over 40 min, *t*_R_ 20.7 min, Alltech-Altima C18 column (10 µm, 22 mm ID, 300 mm, 7 mL/min)) to yield compound **4a** (0.24 g, 72%) as a white foam; **IR** (ATR) 3272, 1634, 1546 cm^−1^; **^1^H NMR** (200 MHz, CDCl_3_) δ 7.51 (1H, s, H^g^), 6.95 (1H, br s, NH), 6.48–6.55 (1H, m, Ar), 6.40–6.48 (1H, m, Ar), 5.80–5.90 (1H, m, Ar), 4.25 (2H, t, *J* 6.2 Hz, H^h^), 3.75 (3H, s, NCH_3_), 3.63 (2H, s, H^f^), 3.16 (2H, m, H^j^), 2.20–2.70 (16H, m, H^a,b,c^), 1.87–2.10 (2H, m, H^i^), 1.58–1.66 (2H, m, H^d^), 1.60–1.58 (2H, m, H^e^) (Figure S13); **^13^C NMR** (75.5 MHz, CDCl_3_) δ 162.0, 143.8, 127.4, 125.1, 122.6, 111.7, 106.7, 54.1, 52.2, 50.4, 49.0, 48.8, 48.3, 47.6, 47.4, 46.8, 46.7, 36.2, 35.7, 30.1, 28.4, 25.7 (Figure S14); **MS** (ESI) m/z 446.3 (MH^+^, 92%), **HRMS** (ESI) calcd for C_22_H_40_N_9_O^+^ 446.33558 found 446.33463 (MH^+^).

#### *N*-(3-4-((1,4,8,11-Tetraazacyclotetradecan-1-yl)methyl)-1*H*-1,2,3-trizol-1-yl)propyl)-1-methyl-4-(1-methyl-1*H*-pyrrole-2-carboxamido)-1*H*-pyrrole-2-carboxamide 4b[81]

Bismethylpyrrole tri-Boc-protected cyclam **3b** (278 mg, 0.32 mmol, 1 eq) was deprotected according to general procedure C yielding compound **4b** (180 mg, 99%) as a white gum without any further purification; **IR** (ATR) 3288, 2935, 1641 cm^−1^; **^1^H NMR** (200 MHz, CDCl_3_) δ 8.75 (1H, br s, NH), 7.65 (1H, s, H^g^), 7.32–7.36 (1H, m, Ar), 6.80–6.90 (2H, m, Ar), 6.70–7.76 (1H, m, Ar), 6.57–6.68 (1H, m, Ar), 6.05–6.13 (1H, m, Ar), 4.40–4.50 (2H, m, H^h^), 3.96 (3H, s, NCH_3_), 3.87 (3H, s, NCH_3_), 3.65–3.70 (2H, m, H^f^), 3.36–3.43 (2H, m, H^j^), 2.70–2.85 (12H, m, H^a^), 2.64–2.70 (2H, m, H^b^), 2.50–2.63 (2H, m, H^c^), 2.15–2.25 (2H, m, H^i^), 1.80–1.90 (2H, m, H^d^), 1.65–1.75 (2H, m, H^e^) (Figure S15); **^13^C NMR** (50.3 MHz, CDCl_3_) 161.7, 159.4, 144.4, 128.0, 125.4, 123.0, 121.7, 119.1, 122.2, 106.9, 104.6 103.5, 54.5, 53.4, 53.1, 52.6, 50.2, 49.0, 48.4, 48.0, 47.2, 46.6, 36.4, 36.2, 29.4, 27.1, 25.0 (Figure S16); **MS** (ESI) m/z 568.3 (MH^+^, 100%); **HRMS** (ESI) calcd for C_28_H_46_N_11_O_2_^+^ 568.38359 found 568.38305 (MH^+^).

#### *N*-(3-(4-((1,4,8,11-Tetraazacyclotetradecan-1yl)methyl)-1*H*-1,2,3-triazol-1-yl)propyl)-1-methyl-4-(1-methyl-4-(1-methyl-1*H*-pyrrole-2-carboxamido)-1*H*-pyrrole-2-carboxamido)-1*H*-pyrrole-2-carboxamide 4c[81]

Three-methylpyrrole tri-Boc-protected cyclam **3c** (70 mg, 0.074 mmol, 1 eq) was deprotected according to general procedure C yielding compound **4c** (41 mg, 98%) as a pale yellow gum without any further purification; **IR** (ATR) 3267, 2924, 1635 cm^−1^; **^1^H NMR** (300 MHz, CDCl_3_) δ 9.35 (2H, br s, 2 N*H*), 7.67 (1H, s, H^g^), 7.51 (1H, br s, N*H*), 7.42–7.47 (1H, m, Ar), 7.38–7.42 (1H, m, Ar), 7.05–7.16 (1H, m, Ar), 6.88–6.95 (1H, m, Ar), 6.81–6.88 (1H, m, Ar), 6.65–6.75 (1H, m, Ar), 5.97–6.06 (1H, m, Ar), 5.00–5.85 (3H, br s, NH), 4.30–4.40 (2H, m, H^h^), 3.93 (3H, s, NCH_3_), 3.88 (3H, s, NCH_3_), 3.81 (3H, s, NCH_3_), 3.48–3.58 (2H, m, H^f^), 3.27–3.40 (2H, m, H^j^), 2.65–2.90 (12H, m, H^a^), 2.51–2.62 (2H, m, H^b^), 2.43–2.51 (2H, m, H^c^), 2.10–2.20 (2H, m, H^i^), 1.72–1.80 (2H, m, H^d^), 1.60–1.72 (2H, m, H^e^) (Figure S17); **^13^C NMR** (75.5 MHz, CDCl_3_) δ 162.0, 159.3, 158.9, 144.1, 128.1, 125.2, 123.3, 122.3, 122.2, 122.0, 121.8, 119.3, 118.8, 114.7, 112.8, 107.1, 103.6, 54.3, 50.0, 48.8, 48.5, 48.2, 47.7, 47.2, 46.1, 45.3, 36.9, 36.6, 36.5, 29.6, 29.2, 23.9 (Figure S18); **MS** (ESI) m/z 690.3 (MH^+^, 100%), **HRMS** (ESI) calcd for C_34_H_52_N_13_O_3_^+^ 690.43161 found 690.43152 (MH^+^).

Complex **5a**. Copper(II) chloride was complexed with **4a** (2.0 mg, 4.50 µmol, 1.0 eq) according to general procedure D. The solution was made up to 3 mL in methanol to a final concentration of 1.50 mM; **UV-vis** (MeOH) λ_max_ = 590 nm, ε = 414 M^−1^ cm^−1^; **MS** (ESI) m/z 543.0 (C_22_H_39_^35^ClCuN_9_O^+^, 100%).

Complex **6a**. Zinc(II) chloride was complexed with **4a** (3.2 mg, 7.2 µmol, 1.0 eq) according to general procedure D. **MS** (ESI) m/z 581.0 (multiplet). ^1^H NMR spectrum shown as Figure S19.

Complex **5b**. Copper(II) chloride was complexed with **4b** (8.8 mg, 15.5 µmol, 1.0 eq) according to procedure D. The solution was made up to 3 mL in methanol to a final concentration of 5.2 mM; **UV-vis** (MeOH) λ_max_ = 615 nm, ε = 113.8 M^−1^ cm^−1^; **MS** (ESI) m/z 665.3 (C_28_H_45_^35^ClCuN_11_O_2_^+^, 100%), 667.3 (C_28_H_45_^37^ClCuN_11_O_2_^+^, 86%); **HRMS** (ESI) calcd for C_28_H_45_^35^ClCuN_11_O_2_^+^ 665.27422 found 665.27305 ((M-Cl)^+^), calcd for C_28_H_45_^37^ClCuN_11_O_2_^+^ 667.27242 found 667.27176 ((M-Cl)^+^).

Complex **6b**. Zinc(II) chloride was complexed with **4b** (2.7 mg, 4.8 µmol, 1.0 eq) according to procedure D. **MS** (ESI) m/z 583.3 (100%). ^1^H NMR spectrum shown as Figure S20.

Complex **5c**. Copper(II) chloride was complexed with **4c** (0.94 mg, 1.36 µmol, 1.0 eq) according to procedure D. The solution was made up to 3 mL in to a final concentration of 0.45 mM; **UV-vis** (MeOH) λ_max_ = 615 nm, ε = 162.7 M^−1^ cm^−1^; **IR** (ATR) 3446, 2925, 1640, 1548, 1414, 1254, 1114, 742 cm^−1^; **MS** (ESI) m/z 543.0 (C_22_H_39_^35^ClCuN_9_O^+^, 100%).

Complex **6c**. Zinc(II) chloride was complexed with **4c** (1.5 mg, 2.2 µmol, 1.0 eq) according to procedure D. **MS** (ESI) m/z 876.0 (96%), 875.1 (100%), 797.4 (93%), 795.3 (82%). ^1^H NMR spectrum shown as Figure S21.

## Supporting Information

Scheme S1 Synthetic Scheme for Supporting Information Compounds.
(TIF)

Figure S1 CDCl_3_, 400 MHz ^1^H NMR spectrum of *N*-(3-azidopropyl)-1-methylpyrrole-2-carboxamide (**2a**).
(TIFF)

Figure S2 CDCl_3_, 50.3 MHz ^13^C NMR spectrum of *N*-(3-azidopropyl)-1-methylpyrrole-2-carboxamide (**2a**).
(TIFF)

Figure S3 CDCl_3_, 300 MHz ^1^H NMR spectrum of *N*-(3-azidopropyl)-1-methyl-4-(1-methyl-1*H*-pyrrole-2-carboxamido)-1*H*-pyrrole-2-carboxamide (**2b**).
(TIFF)

Figure S4 CDCl_3_, 75.5 MHz ^13^C NMR spectrum of *N*-(3-azidopropyl)-1-methyl-4-(1-methyl-1*H*-pyrrole-2-carboxamido)-1*H*-pyrrole-2-carboxamide (**2b**).
(TIFF)

Figure S5 CDCl_3_, 300 MHz ^1^H NMR spectrum of *N*-(3-Azidopropyl)-1-methyl-4-(1-methyl-4-(1-methyl-1*H*-pyrrole-2-carboxamido)-1*H*-pyrrole-2-carboxamido)-1*H*-pyrrole-2-carboxamide (**2c**).
(TIFF)

Figure S6 CDCl_3_, 75.5 MHz ^13^C NMR spectrum of *N*-(3-azidopropyl)-1-methyl-4-(1-methyl-4-(1-methyl-1*H*-pyrrole-2-carboxamido)-1*H*-pyrrole-2-carboxamido)-1*H*-pyrrole-2-carboxamide (**2c**).
(TIFF)

Figure S7 CDCl_3_, 300 MHz ^1^H NMR spectrum of tri-*tert*-butyl 11-((1-(3-(1-methyl-1*H*-pyrrole-2-carboxamido)propyl)-1*H*-1,2,3-triazol-4-yl)methyl)-1,4,8,11-tetraazacyclotetradecane-1,4,8-tricarboxylate (**3a**).
(TIFF)

Figure S8 CDCl_3_, 50.3 MHz ^13^H NMR spectrum of Tri-*tert*-butyl 11-((1-(3-(1-methyl-1*H*-pyrrole-2-carboxamido)propyl)-1*H*-1,2,3-triazol-4-yl)methyl)-1,4,8,11-tetraazacyclotetradecane-1,4,8-tricarboxylate (**3a**).
(TIFF)

Figure S9 CDCl_3_, 300 MHz ^1^H NMR spectrum of tri-*tert*-butyl 11-((1-(3-(1-methyl-4-(1-methyl-1*H*-pyrrole-2-carboxamido)-1*H*-pyrrole-2-carboxamido)propyl)-1*H*-1,2,3-triazol-4-yl)methyl)-1,4,8,11-tetraazacyclotetradecane-1,4,8-tricarboxylate (**3b**).
(TIFF)

Figure S10 CDCl_3_, 50.3 MHz ^13^C NMR spectrum of tri-*tert*-butyl 11-((1-(3-(1-methyl-4-(1-methyl-1*H*-pyrrole-2-carboxamido)-1*H*-pyrrole-2-carboxamido)propyl)-1*H*-1,2,3-triazol-4-yl)methyl)-1,4,8,11-tetraazacyclotetradecane-1,4,8-tricarboxylate (**3b**).
(TIFF)

Figure S11 CDCl_3_, 300 MHz ^1^H NMR spectrum of tri-*tert*-butyl 11-((1-(3-(1-methyl-4-(1-methyl-4-(1-methyl-1*H*-pyrrole-2-carboxamido)-1*H*-pyrrole-2-carboxamido)-1*H*-pyrrole-2-carboxamido)propyl)-1*H*-1,2,3-triazol-4-yl)methyl)-1,4,8,11-tetraazacyclotetradecane-1,4,8-tricarboxylate (**3c**).
(TIFF)

Figure S12 CDCl_3_, 50.3 MHz ^13^C NMR spectrum of tri-*tert*-butyl 11-((1-(3-(1-methyl-4-(1-methyl-4-(1-methyl-1*H*-pyrrole-2-carboxamido)-1*H*-pyrrole-2-carboxamido)-1*H*-pyrrole-2-carboxamido)propyl)-1*H*-1,2,3-triazol-4-yl)methyl)-1,4,8,11-tetraazacyclotetradecane-1,4,8-tricarboxylate (**3c**).
(TIFF)

Figure S13 CDCl_3_, 200 MHz ^1^H NMR spectrum of *N*-(3-(4-((1,4,8,11-tetraazacyclotetradecan-1-yl)methyl)-1*H*-1,2,3-triazol-1-yl)propyl)-1-methyl-1*H*-pyrrole-2-carboxamide (**4a**).
(TIFF)

Figure S14 CDCl_3_, 75.5 MHz ^13^C NMR spectrum of *N*-(3-(4-((1,4,8,11-tetraazacyclotetradecan-1-yl)methyl)-1*H*-1,2,3-triazol-1-yl)propyl)-1-methyl-1*H*-pyrrole-2-carboxamide (**4a**).
(TIFF)

Figure S15 CDCl_3_, 200 MHz ^1^H NMR spectrum of *N*-(3-4-((1,4,8,11-tetraazacyclotetradecan-1-yl)methyl)-1*H*-1,2,3-trizol-1-yl)propyl)-1-methyl-4-(1-methyl-1*H*-pyrrole-2-carboxamido)-1*H*-pyrrole-2-carboxamide (**4b**).
(TIFF)

Figure S16 CDCl_3_, 50.3 MHz ^13^C NMR spectrum of *N*-(3-4-((1,4,8,11-tetraazacyclotetradecan-1-yl)methyl)-1*H*-1,2,3-trizol-1-yl)propyl)-1-methyl-4-(1-methyl-1*H*-pyrrole-2-carboxamido)-1*H*-pyrrole-2-carboxamide (**4b**).
(TIFF)

Figure S17 CDCl_3_, 300 MHz ^1^H NMR spectrum of *N*-(3-(4-((1,4,8,11-tetraazacyclotetradecan-1yl)methyl)-1*H*-1,2,3-triazol-1-yl)propyl)-1-methyl-4-(1-methyl-4-(1-methyl-1*H*-pyrrole-2-carboxamido)-1*H*-pyrrole-2-carboxamido)-1*H*-pyrrole-2-carboxamide (**4c**).
(TIFF)

Figure S18 CDCl_3_, 75.5 MHz ^13^C NMR spectrum of *N*-(3-(4-((1,4,8,11-tetraazacyclotetradecan-1yl)methyl)-1*H*-1,2,3-triazol-1-yl)propyl)-1-methyl-4-(1-methyl-4-(1-methyl-1*H*-pyrrole-2-carboxamido)-1*H*-pyrrole-2-carboxamido)-1*H*-pyrrole-2-carboxamide (**4c**).
(TIFF)

Figure S19 300 MHz, MeOD, ^1^H NMR spectrum of mono-pyrrole zinc chloride cyclam complex (**6a**).
(TIFF)

Figure S20 300 MHz, MeOD, ^1^H NMR spectrum of di-pyrrole zinc chloride cyclam complex (**6b**).
(TIFF)

Figure S21 300 MHz, MeOD, ^1^H NMR spectrum of tri-pyrrole zinc chloride cyclam complex (**6c**).
(TIFF)

Figure S22 [125] Job plot for formation of complex between copper(II) and ligand **4a**.
(TIFF)

Figure S23 UV-vis spectrum for the titration of a solution of CuCl_2_ with compound **4c** in methanol (graphical representation of raw data).
(TIFF)

Figure S24 Example ITC curve for GC-rich oligonucleotide illustrating no observable binding; titration of 1000 µM **4c** to 10 µM GC oligo (oligo **II**).
(TIFF)

Spreadsheet S1 Error calculations for Table 1.
(XLS)

Text S1 Procedures for preparation of known compounds, and description of entropy error calculations.
(DOC)

Table S1 Effect of structural modifications of lexitropsins on binding affinities for compound **4c** *vs*. selected literature compounds.
(DOC)

NMR Data S1 Raw NMR data files (.dx) for compounds **2**–**4** (^1^H and ^13^C) and **6** (^1^H).
(ZIP)
