# Supplementary figures and images for: Polyamide-Scorpion Cyclam Lexitropsins Selectively Bind AT-Rich DNA Independently of the Nature of the Coordinated Metal
Source: PLoS One. 2011 May 9;6(5):e17446. doi: 10.1371/journal.pone.0017446 (PMC3090394; doi:10.1371/journal.pone.0017446)

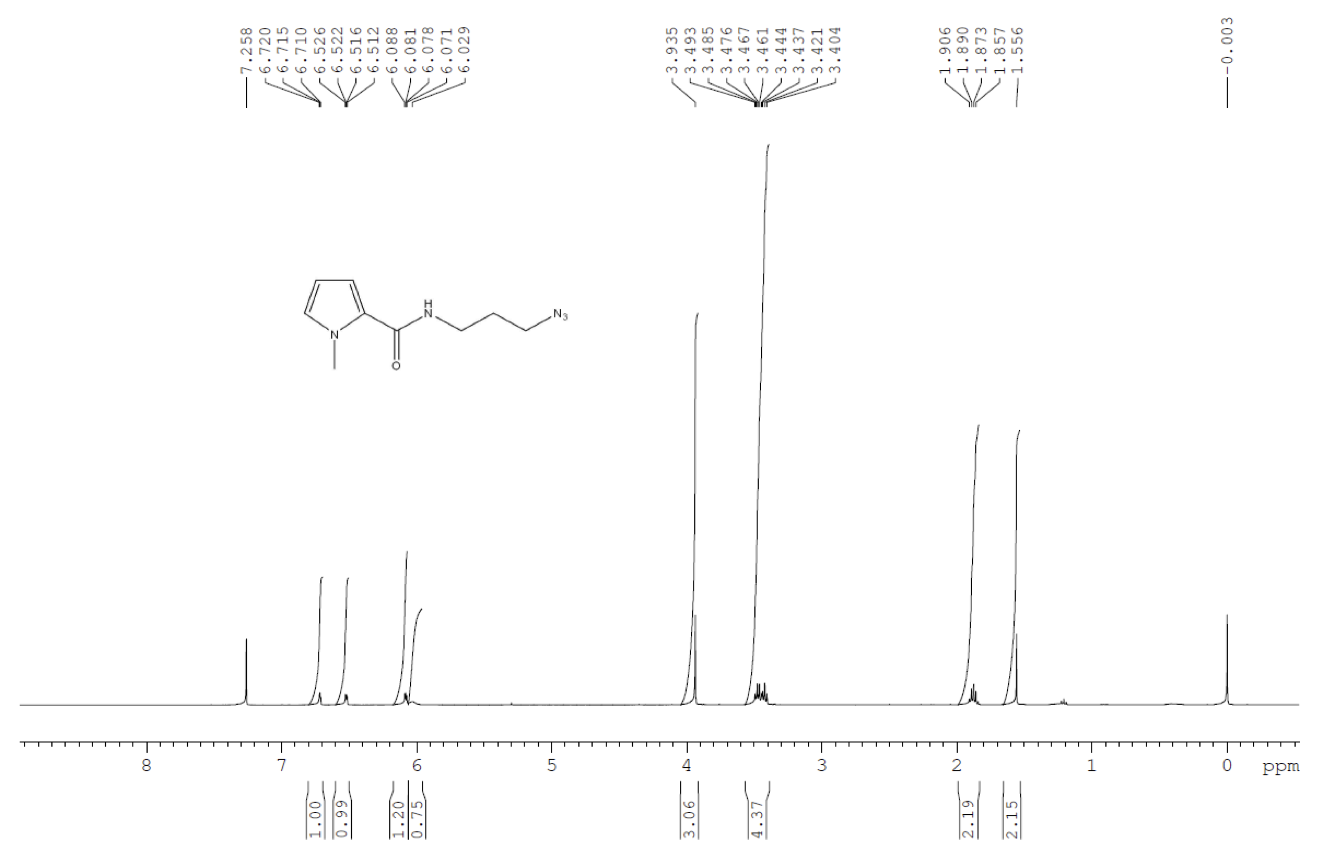

Supplement: Figure S1 — CDCl3, 400 MHz 1H NMR spectrum of N-(3-azidopropyl)-1-methylpyrrole-2-carboxamide (2a). (TIFF) [file pone.0017446.s002.tiff]

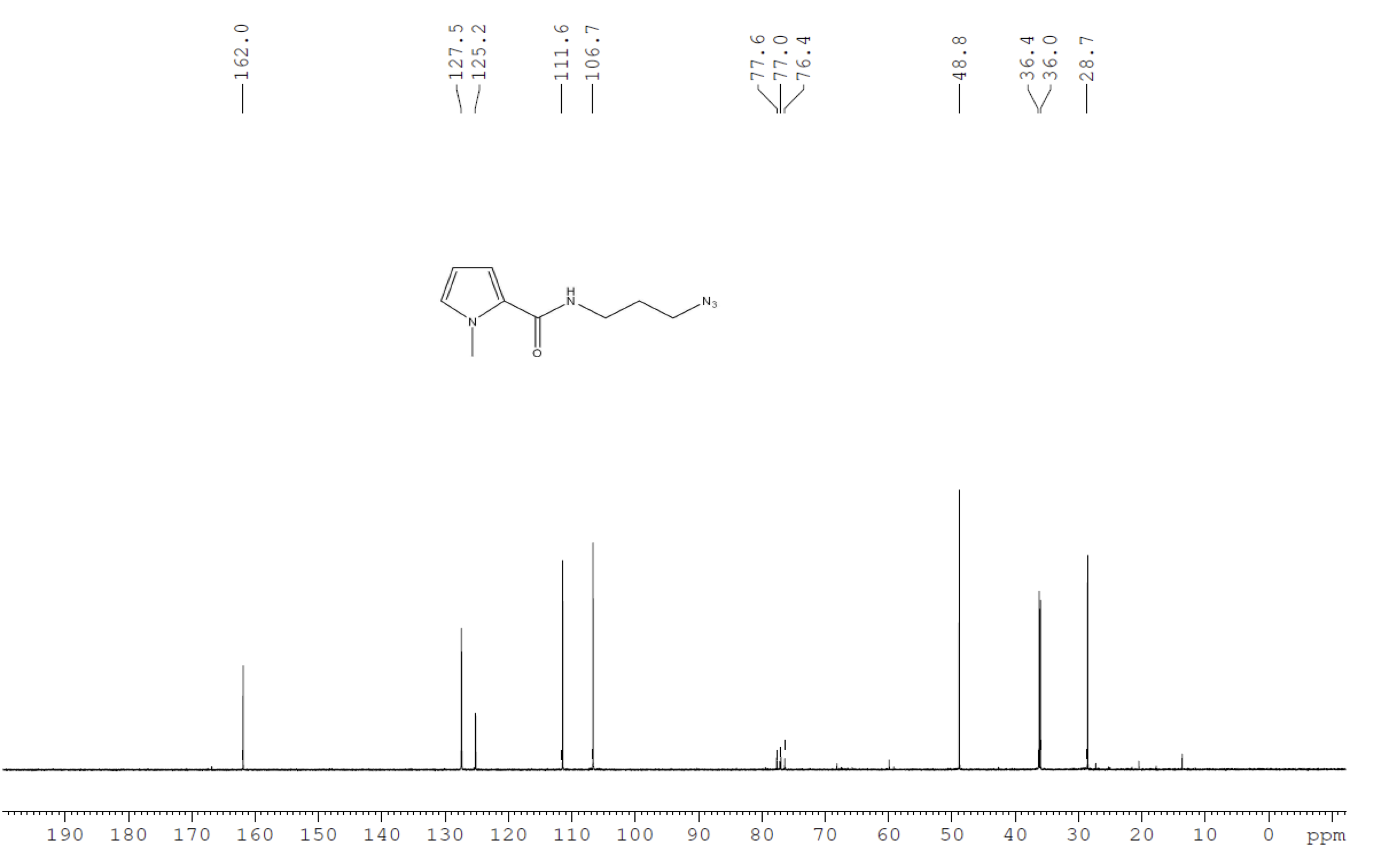

Supplement: Figure S2 — CDCl3, 50.3 MHz 13C NMR spectrum of N-(3-azidopropyl)-1-methylpyrrole-2-carboxamide (2a). (TIFF) [file pone.0017446.s003.tiff]

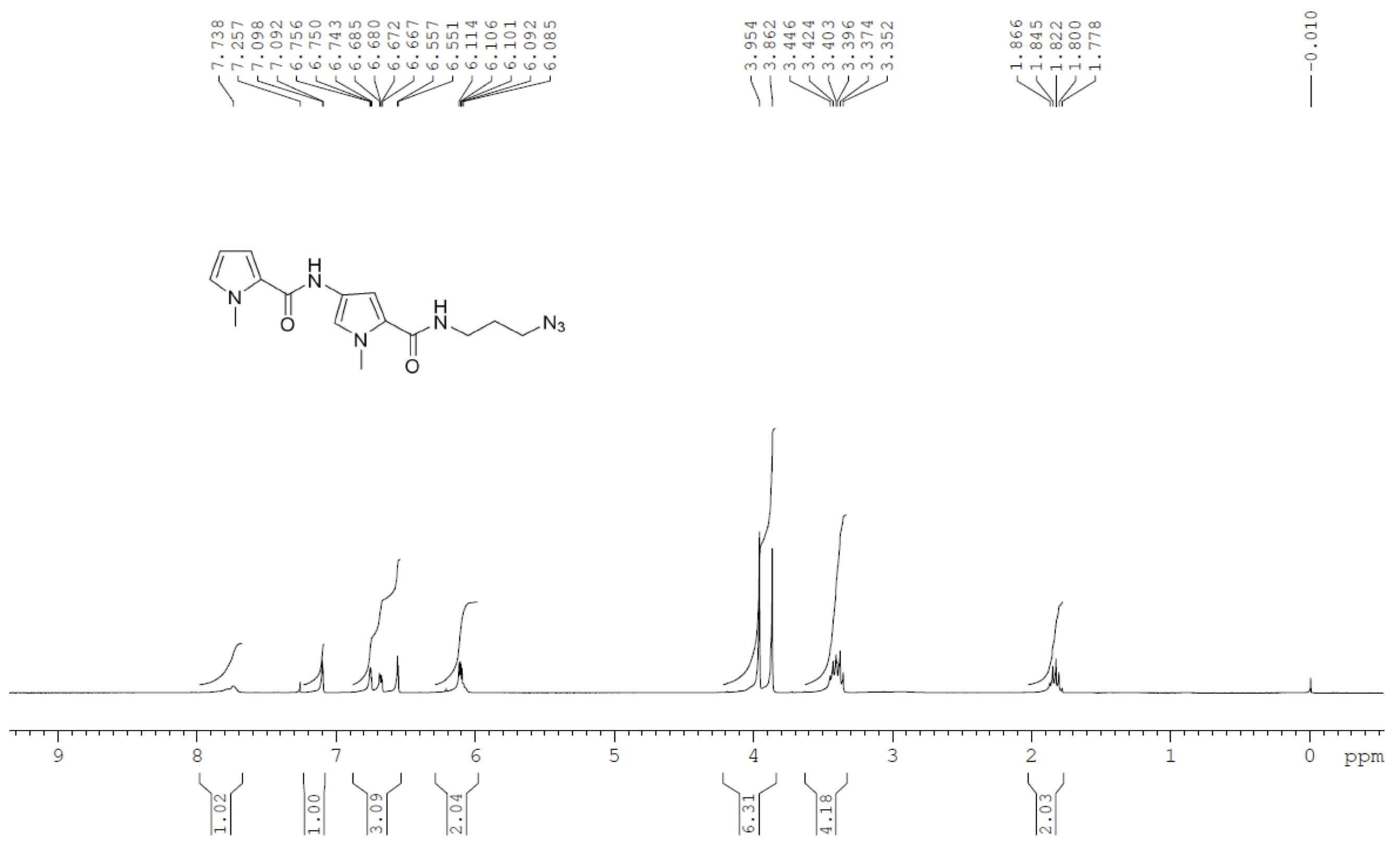

Supplement: Figure S3 — CDCl3, 300 MHz 1H NMR spectrum of N-(3-azidopropyl)-1-methyl-4-(1-methyl-1H-pyrrole-2-carboxamido)-1H-pyrrole-2-carboxamide (2b). (TIFF) [file pone.0017446.s004.tiff]

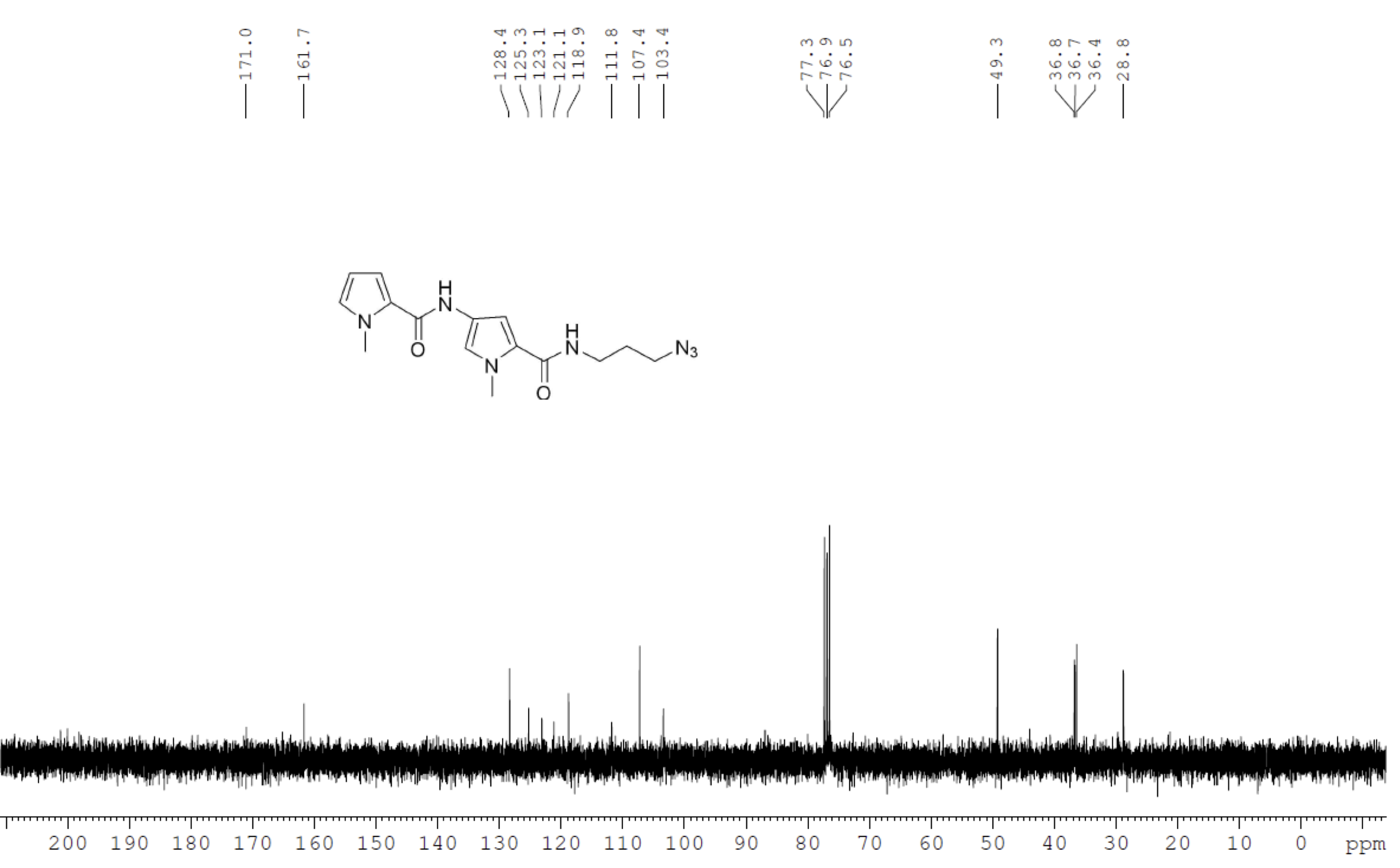

Supplement: Figure S4 — CDCl3, 75.5 MHz 13C NMR spectrum of N-(3-azidopropyl)-1-methyl-4-(1-methyl-1H-pyrrole-2-carboxamido)-1H-pyrrole-2-carboxamide (2b). (TIFF) [file pone.0017446.s005.tiff]

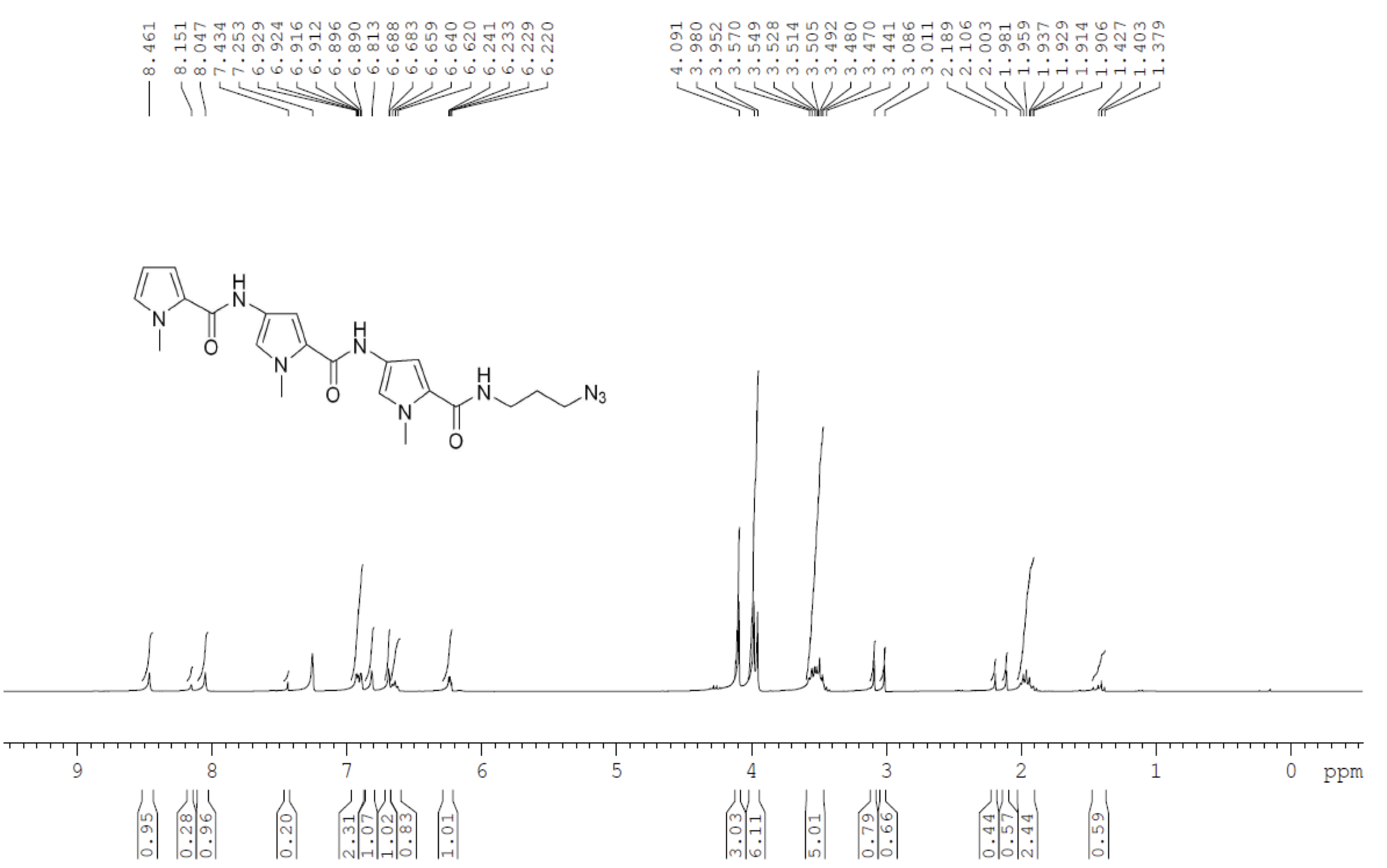

Supplement: Figure S5 — CDCl3, 300 MHz 1H NMR spectrum of N-(3-Azidopropyl)-1-methyl-4-(1-methyl-4-(1-methyl-1H-pyrrole-2-carboxamido)-1H-pyrrole-2-carboxamido)-1H-pyrrole-2-carboxamide (2c). (TIFF) [file pone.0017446.s006.tiff]

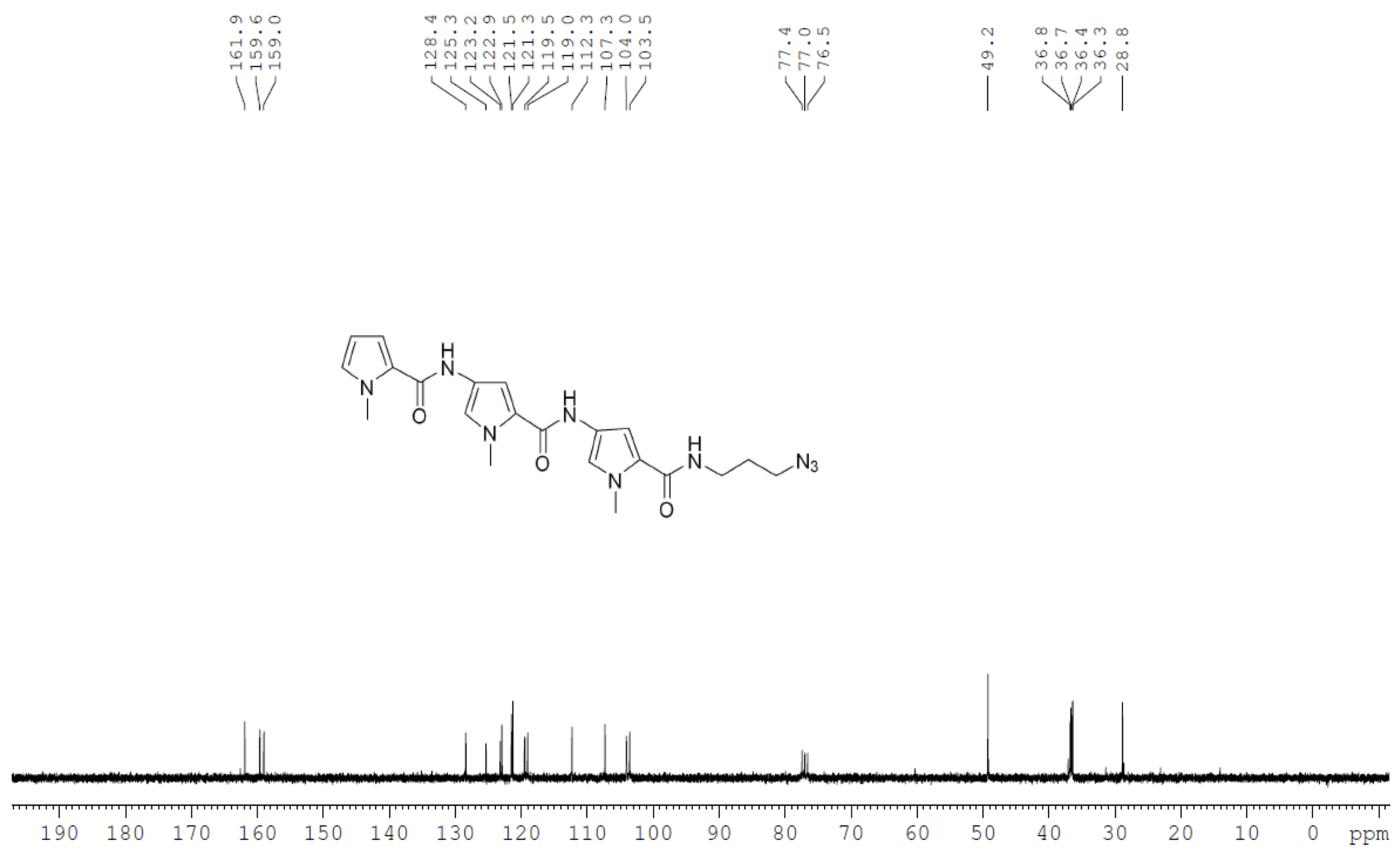

Supplement: Figure S6 — CDCl3, 75.5 MHz 13C NMR spectrum of N-(3-azidopropyl)-1-methyl-4-(1-methyl-4-(1-methyl-1H-pyrrole-2-carboxamido)-1H-pyrrole-2-carboxamido)-1H-pyrrole-2-carboxamide (2c). (TIFF) [file pone.0017446.s007.tiff]

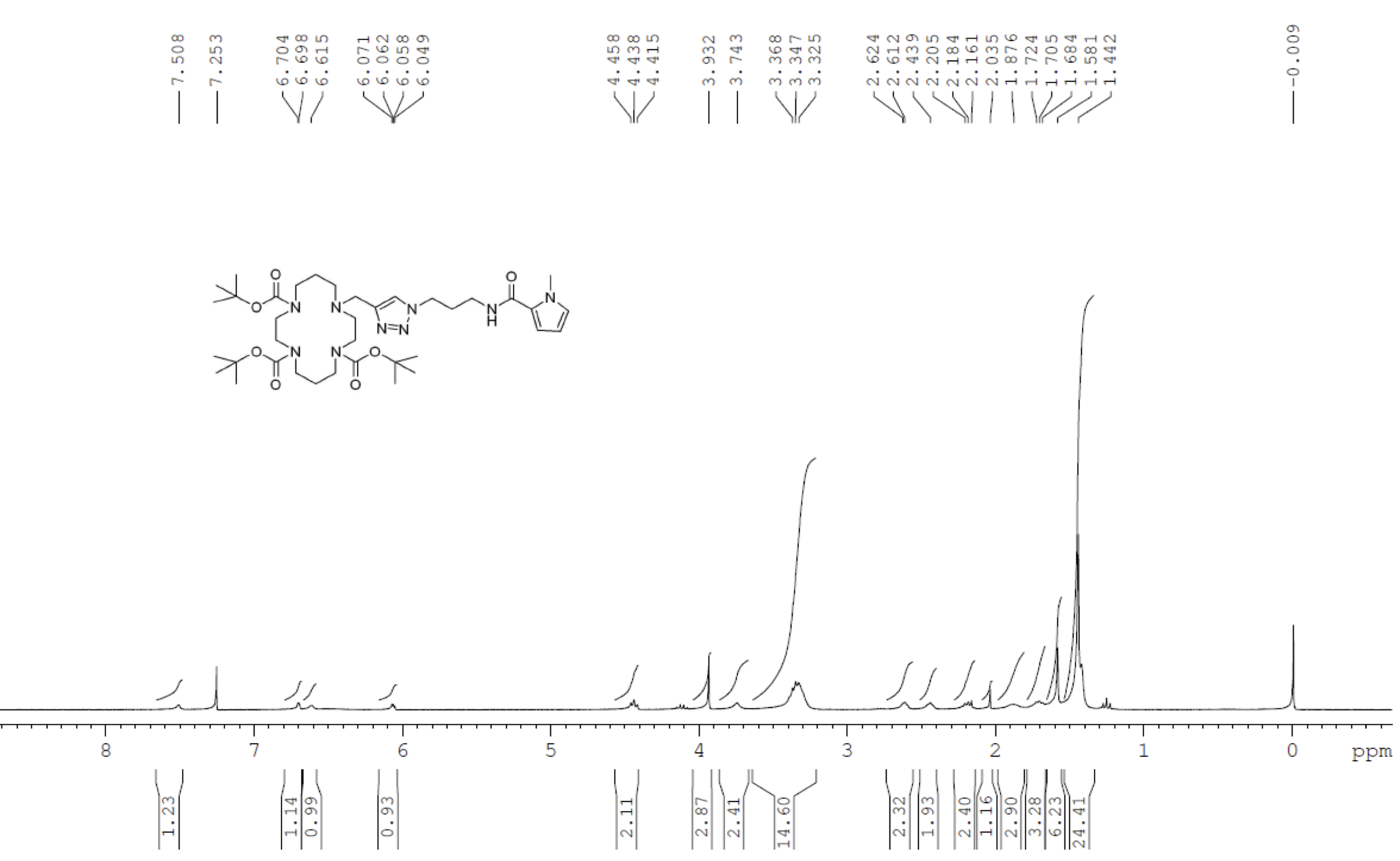

Supplement: Figure S7 — CDCl3, 300 MHz 1H NMR spectrum of tri-tert-butyl 11-((1-(3-(1-methyl-1H-pyrrole-2-carboxamido)propyl)-1H-1,2,3-triazol-4-yl)methyl)-1,4,8,11-tetraazacyclotetradecane-1,4,8-tricarboxylate (3a). (TIFF) [file pone.0017446.s008.tiff]

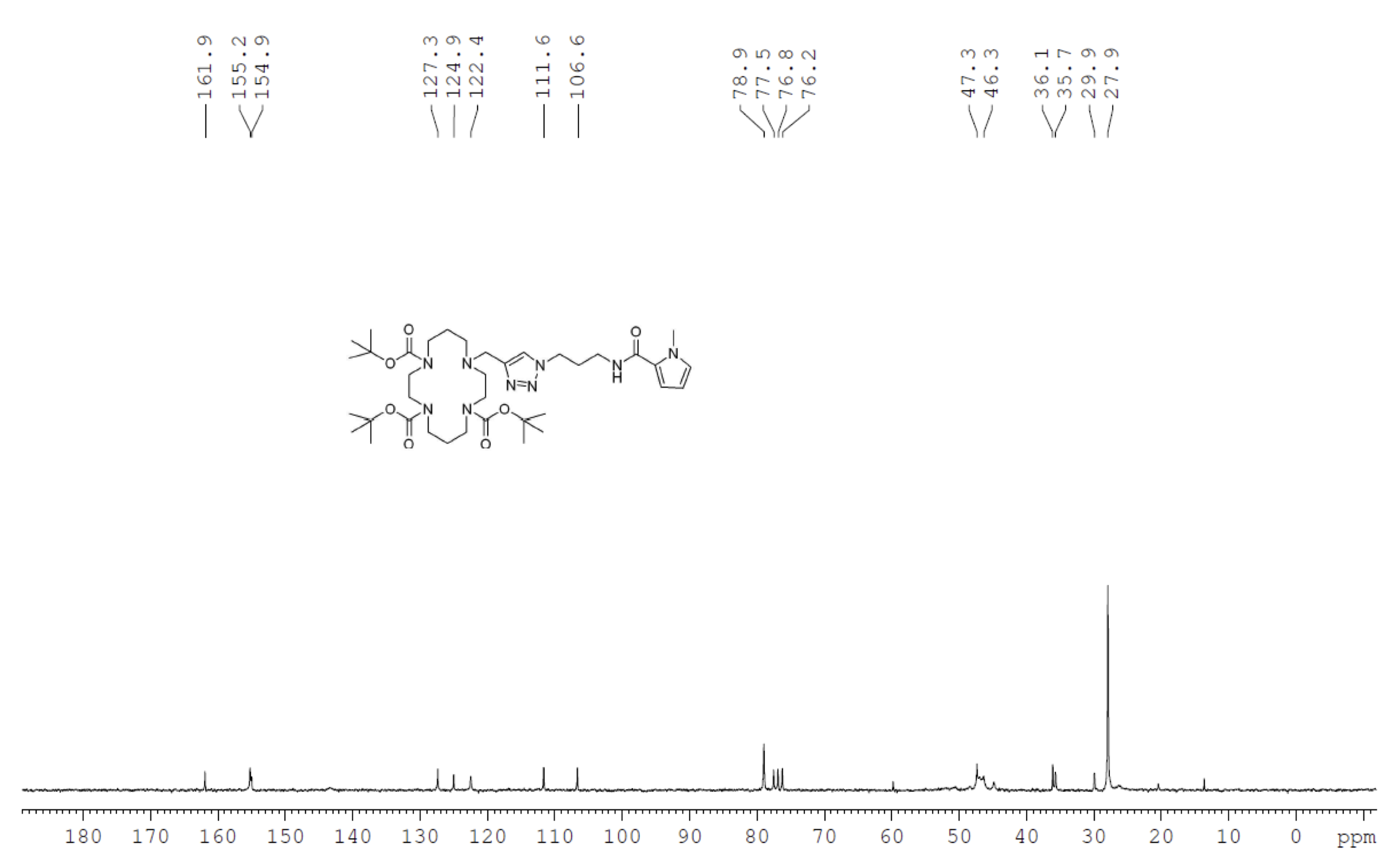

Supplement: Figure S8 — CDCl3, 50.3 MHz 13H NMR spectrum of Tri-tert-butyl 11-((1-(3-(1-methyl-1H-pyrrole-2-carboxamido)propyl)-1H-1,2,3-triazol-4-yl)methyl)-1,4,8,11-tetraazacyclotetradecane-1,4,8-tricarboxylate (3a). (TIFF) [file pone.0017446.s009.tiff]

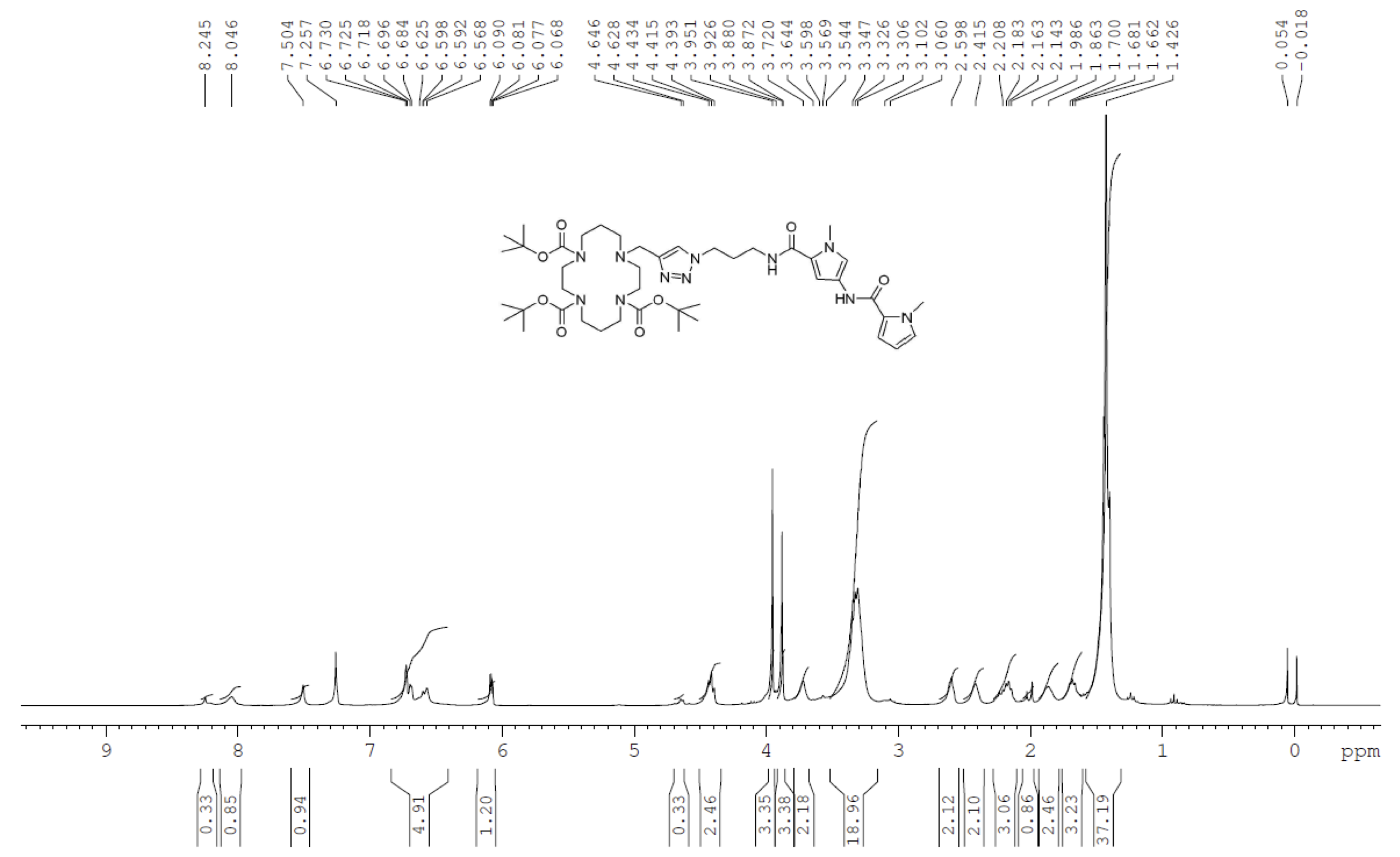

Supplement: Figure S9 — CDCl3, 300 MHz 1H NMR spectrum of tri-tert-butyl 11-((1-(3-(1-methyl-4-(1-methyl-1H-pyrrole-2-carboxamido)-1H-pyrrole-2-carboxamido)propyl)-1H-1,2,3-triazol-4-yl)methyl)-1,4,8,11-tetraazacyclotetradecane-1,4,8-tricarboxylate (3b). (TIFF) [file pone.0017446.s010.tiff]

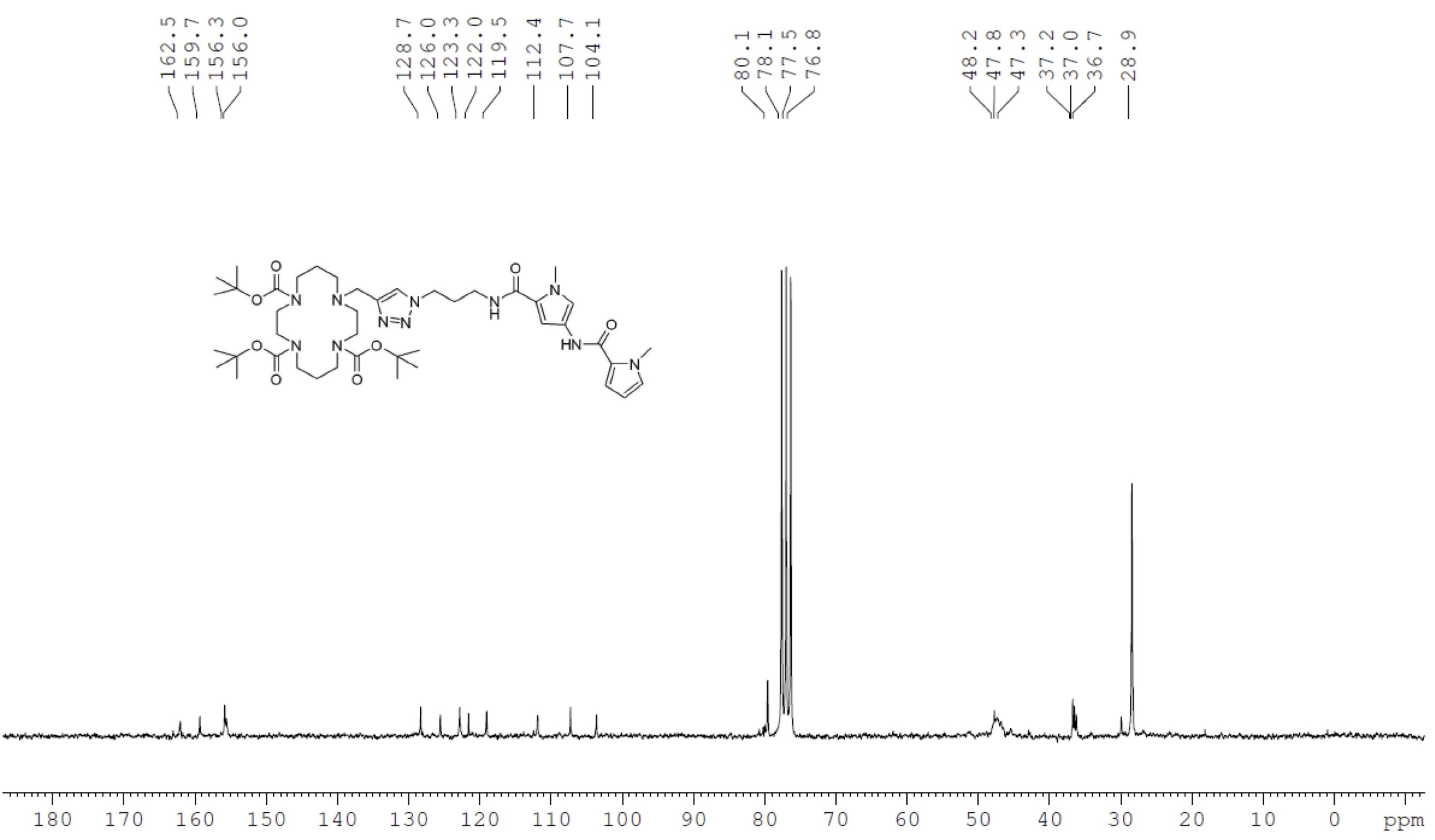

Supplement: Figure S10 — CDCl3, 50.3 MHz 13C NMR spectrum of tri-tert-butyl 11-((1-(3-(1-methyl-4-(1-methyl-1H-pyrrole-2-carboxamido)-1H-pyrrole-2-carboxamido)propyl)-1H-1,2,3-triazol-4-yl)methyl)-1,4,8,11-tetraazacyclotetradecane-1,4,8-tricarboxylate (3b). (TIFF) [file pone.0017446.s011.tiff]

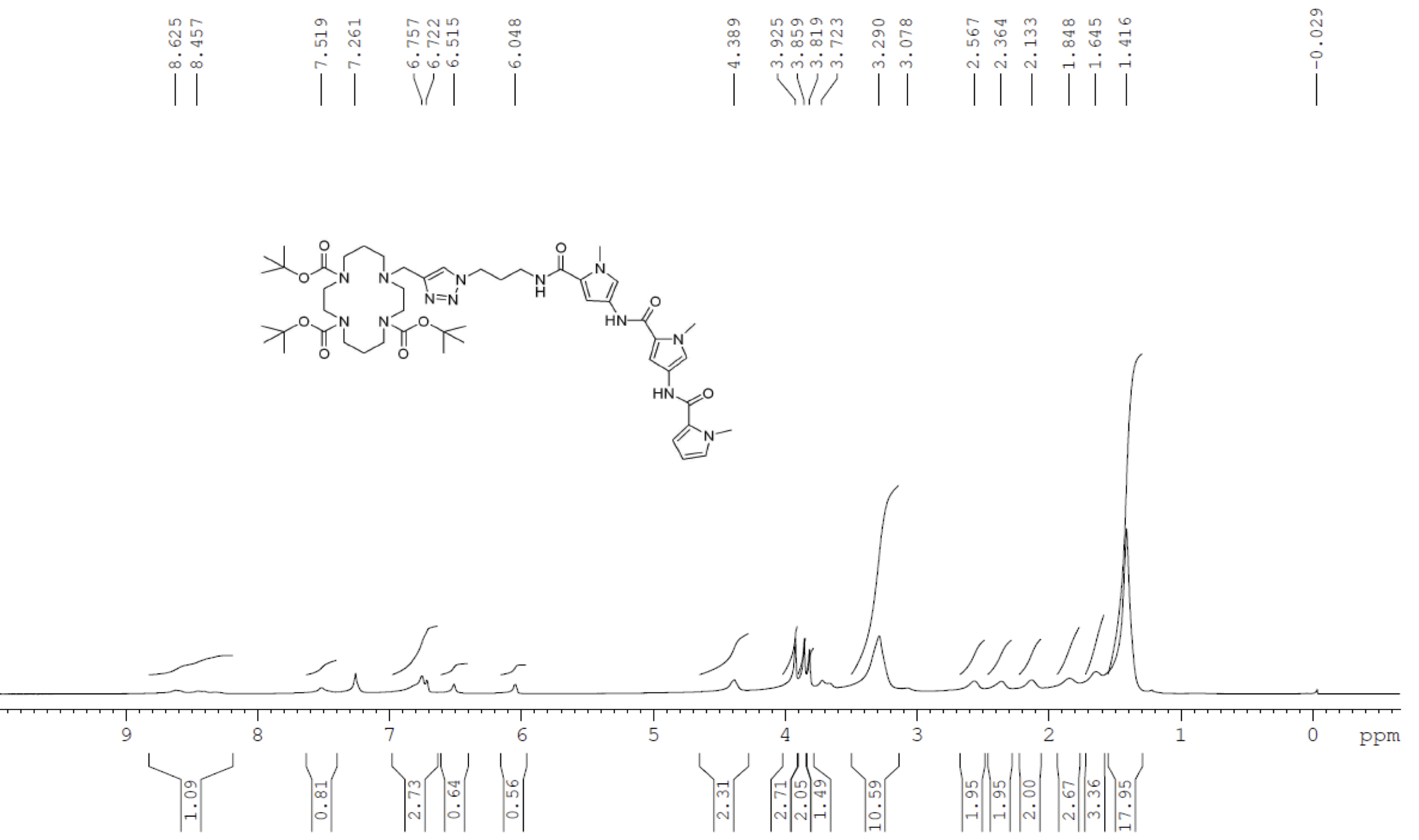

Supplement: Figure S11 — CDCl3, 300 MHz 1H NMR spectrum of tri-tert-butyl 11-((1-(3-(1-methyl-4-(1-methyl-4-(1-methyl-1H-pyrrole-2-carboxamido)-1H-pyrrole-2-carboxamido)-1H-pyrrole-2-carboxamido)propyl)-1H-1,2,3-triazol-4-yl)methyl)-1,4,8,11-tetraazacyclotetradecane-1,4,8-tricarboxylate (3c). (TIFF) [file pone.0017446.s012.tiff]

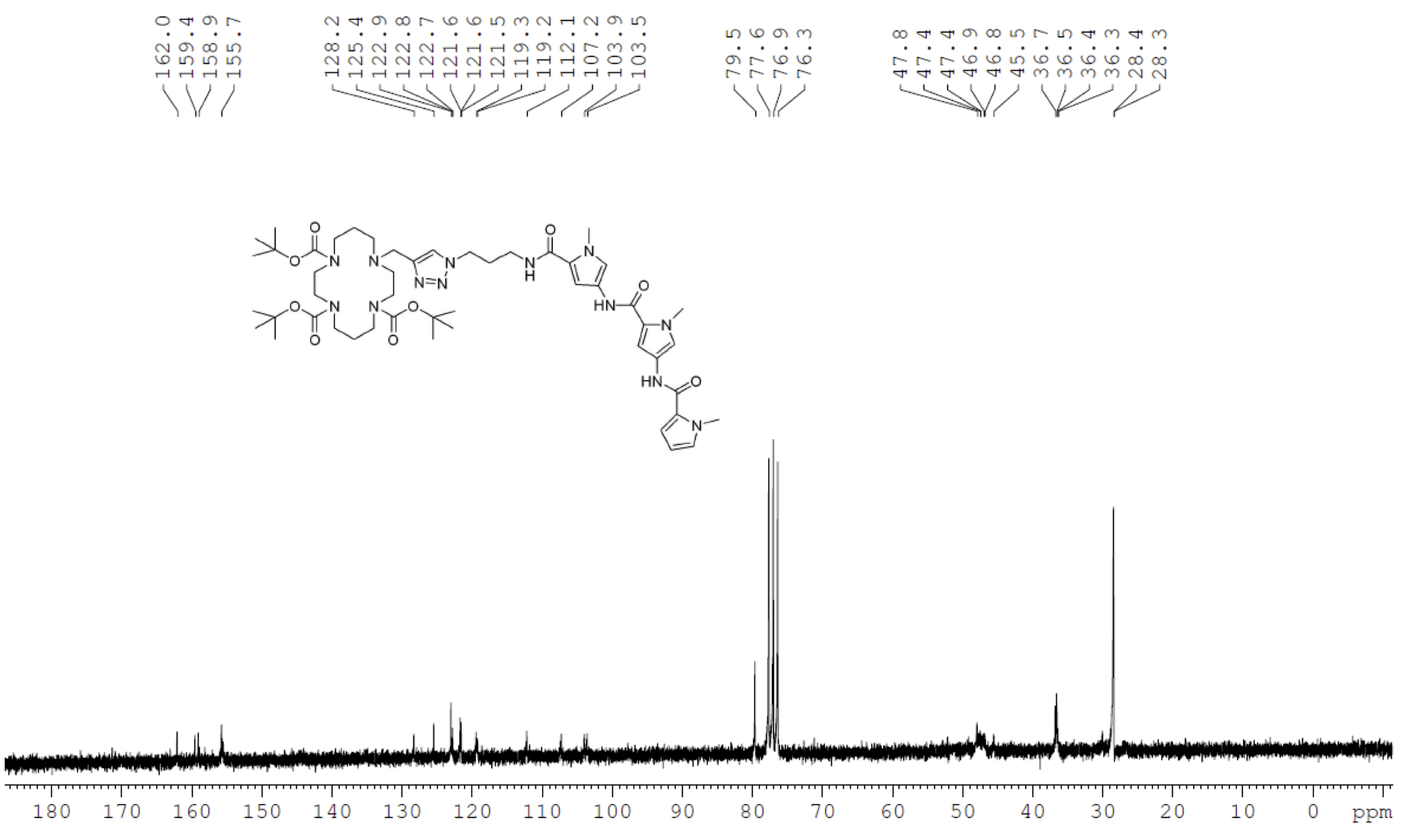

Supplement: Figure S12 — CDCl3, 50.3 MHz 13C NMR spectrum of tri-tert-butyl 11-((1-(3-(1-methyl-4-(1-methyl-4-(1-methyl-1H-pyrrole-2-carboxamido)-1H-pyrrole-2-carboxamido)-1H-pyrrole-2-carboxamido)propyl)-1H-1,2,3-triazol-4-yl)methyl)-1,4,8,11-tetraazacyclotetradecane-1,4,8-tricarboxylate (3c). (TIFF) [file pone.0017446.s013.tiff]

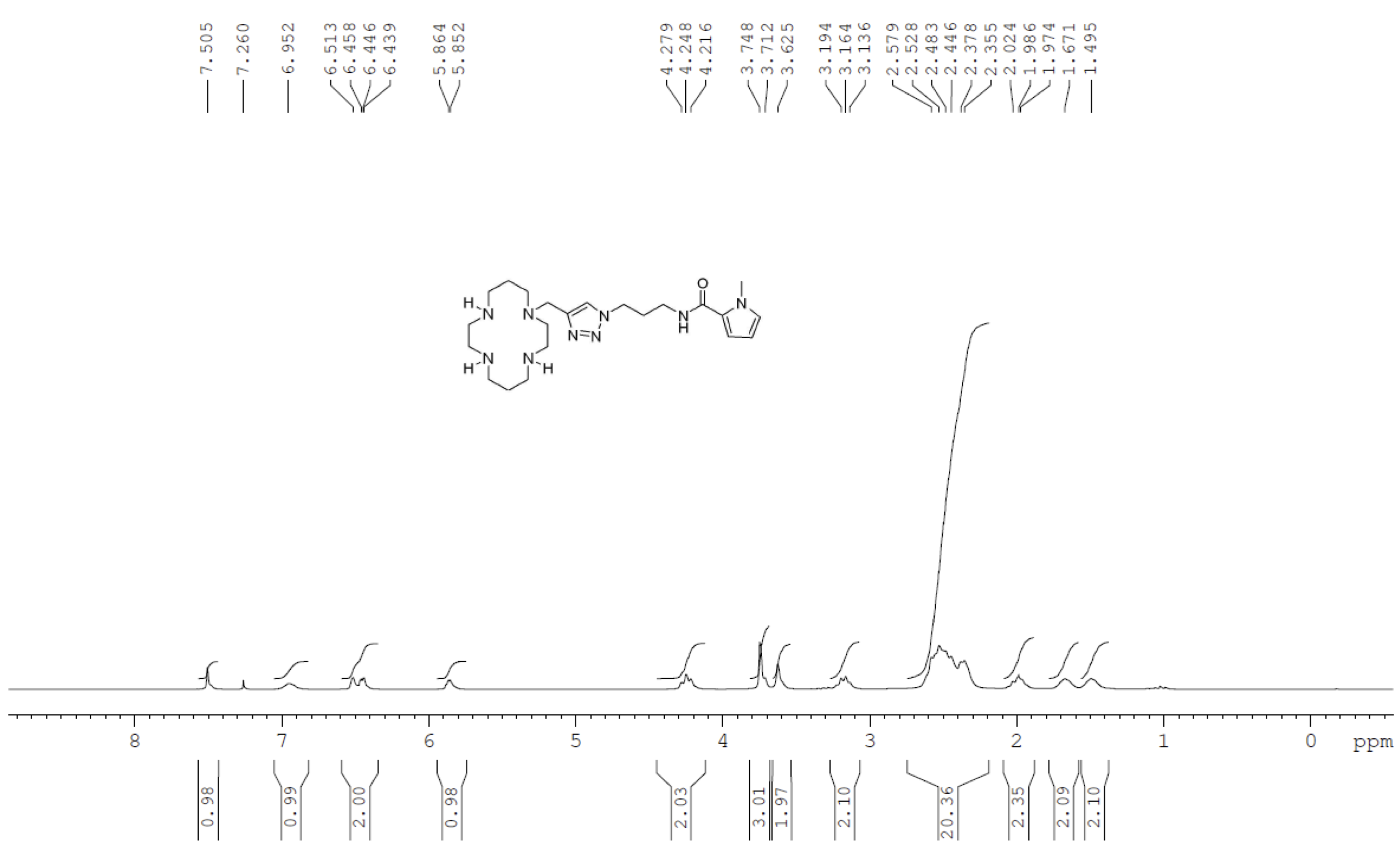

Supplement: Figure S13 — CDCl3, 200 MHz 1H NMR spectrum of N-(3-(4-((1,4,8,11-tetraazacyclotetradecan-1-yl)methyl)-1H-1,2,3-triazol-1-yl)propyl)-1-methyl-1H-pyrrole-2-carboxamide (4a). (TIFF) [file pone.0017446.s014.tiff]

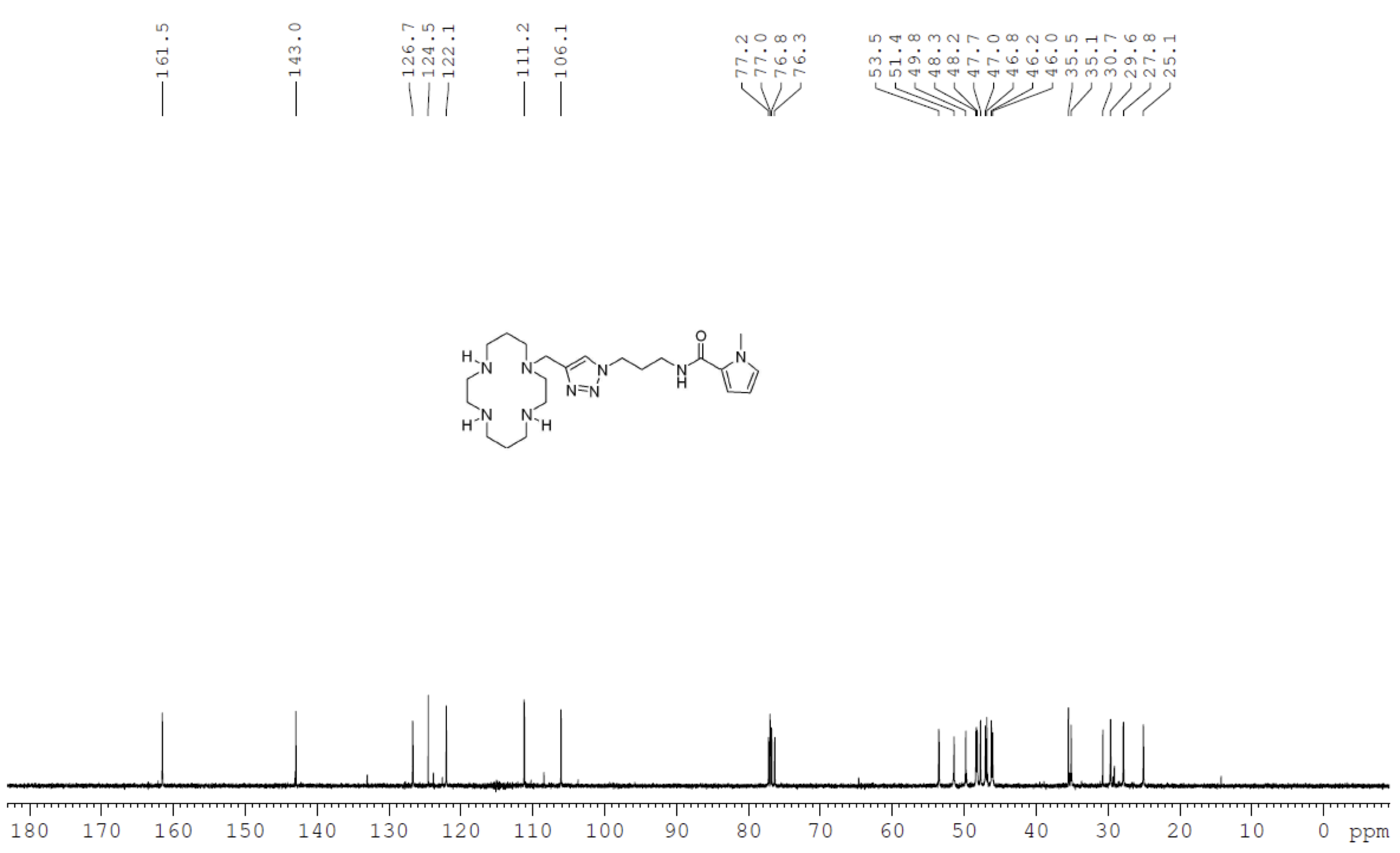

Supplement: Figure S14 — CDCl3, 75.5 MHz 13C NMR spectrum of N-(3-(4-((1,4,8,11-tetraazacyclotetradecan-1-yl)methyl)-1H-1,2,3-triazol-1-yl)propyl)-1-methyl-1H-pyrrole-2-carboxamide (4a). (TIFF) [file pone.0017446.s015.tiff]

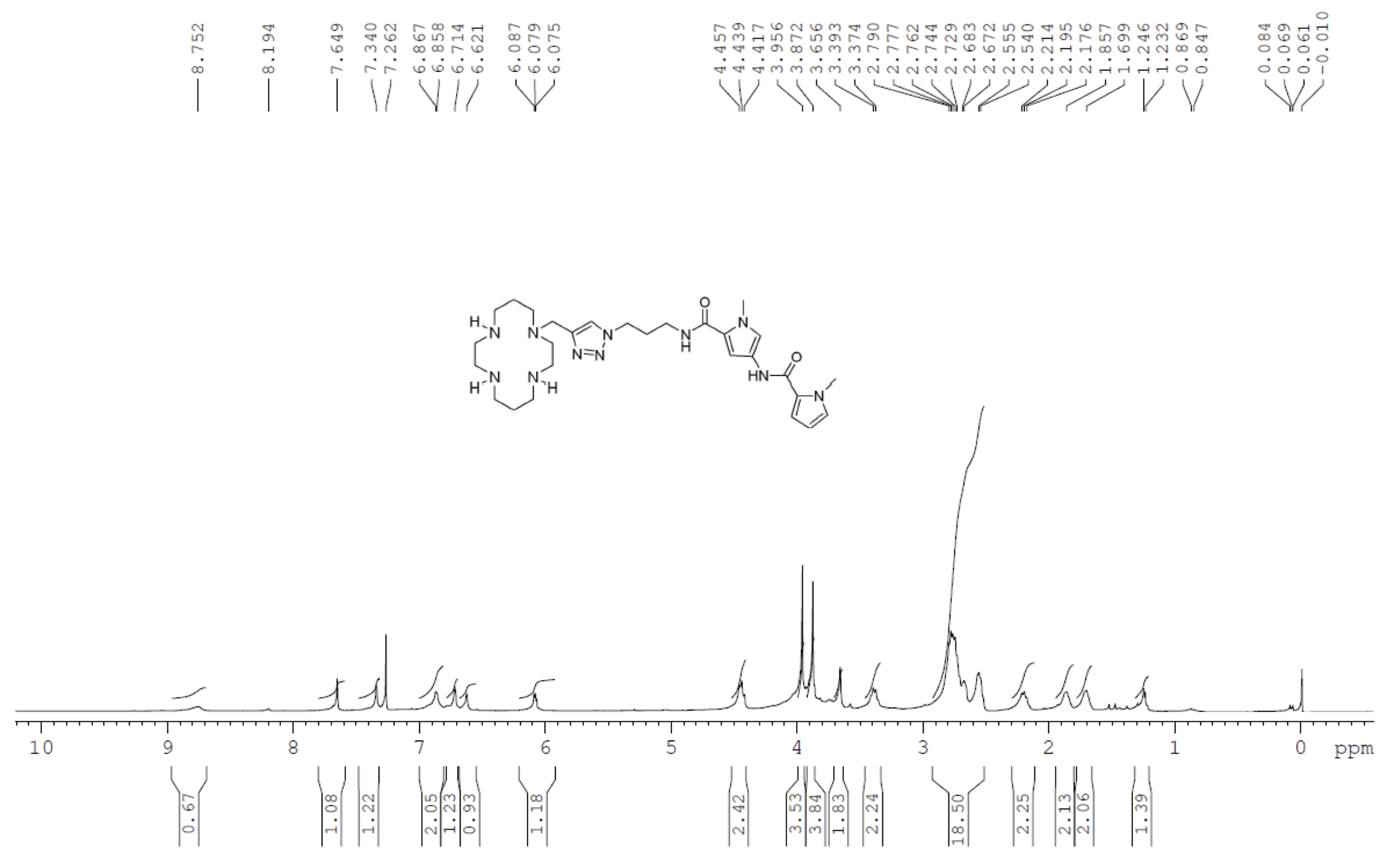

Supplement: Figure S15 — CDCl3, 200 MHz 1H NMR spectrum of N-(3-4-((1,4,8,11-tetraazacyclotetradecan-1-yl)methyl)-1H-1,2,3-trizol-1-yl)propyl)-1-methyl-4-(1-methyl-1H-pyrrole-2-carboxamido)-1H-pyrrole-2-carboxamide (4b). (TIFF) [file pone.0017446.s016.tiff]

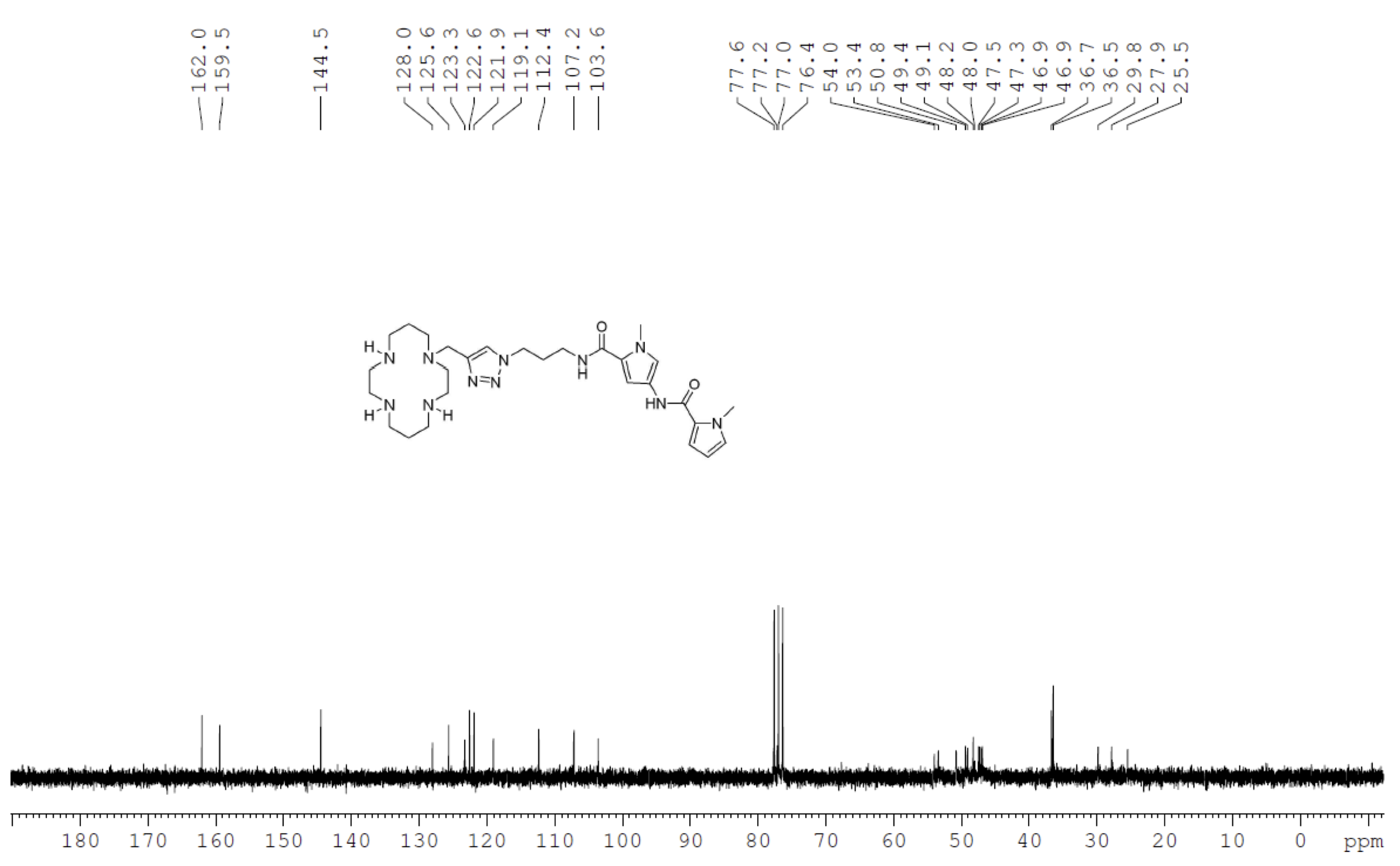

Supplement: Figure S16 — CDCl3, 50.3 MHz 13C NMR spectrum of N-(3-4-((1,4,8,11-tetraazacyclotetradecan-1-yl)methyl)-1H-1,2,3-trizol-1-yl)propyl)-1-methyl-4-(1-methyl-1H-pyrrole-2-carboxamido)-1H-pyrrole-2-carboxamide (4b). (TIFF) [file pone.0017446.s017.tiff]

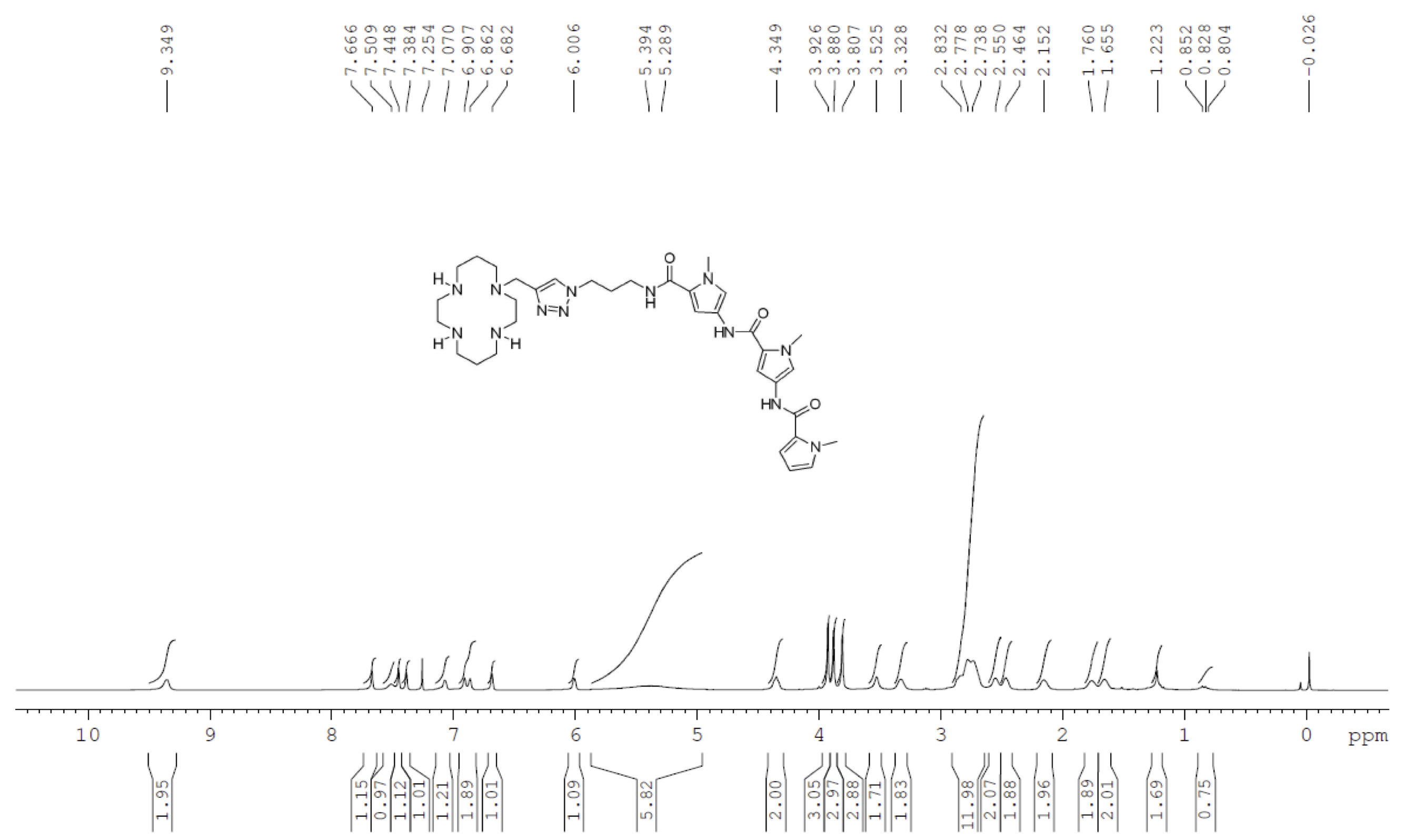

Supplement: Figure S17 — CDCl3, 300 MHz 1H NMR spectrum of N-(3-(4-((1,4,8,11-tetraazacyclotetradecan-1yl)methyl)-1H-1,2,3-triazol-1-yl)propyl)-1-methyl-4-(1-methyl-4-(1-methyl-1H-pyrrole-2-carboxamido)-1H-pyrrole-2-carboxamido)-1H-pyrrole-2-carboxamide (4c). (TIFF) [file pone.0017446.s018.tiff]

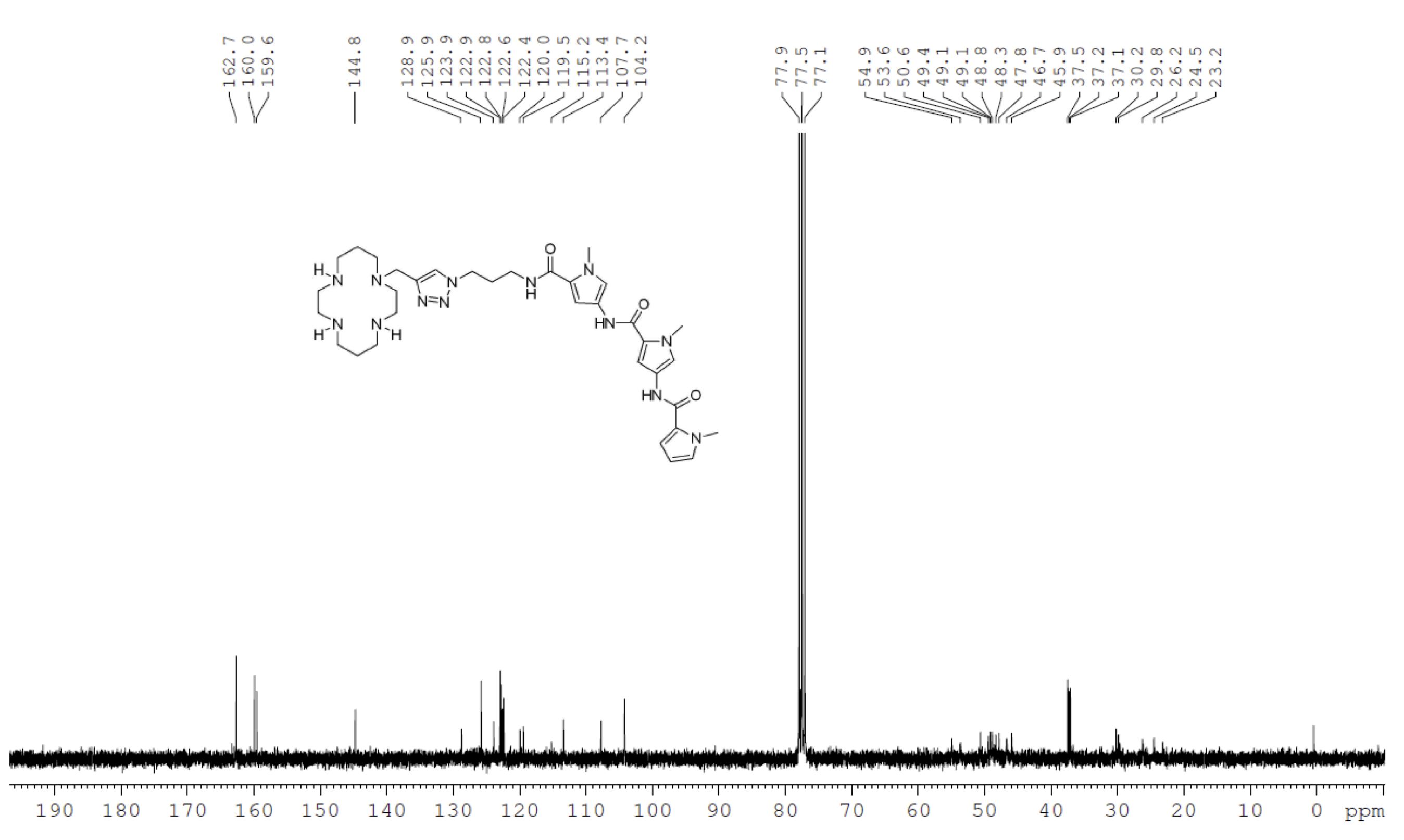

Supplement: Figure S18 — CDCl3, 75.5 MHz 13C NMR spectrum of N-(3-(4-((1,4,8,11-tetraazacyclotetradecan-1yl)methyl)-1H-1,2,3-triazol-1-yl)propyl)-1-methyl-4-(1-methyl-4-(1-methyl-1H-pyrrole-2-carboxamido)-1H-pyrrole-2-carboxamido)-1H-pyrrole-2-carboxamide (4c). (TIFF) [file pone.0017446.s019.tiff]

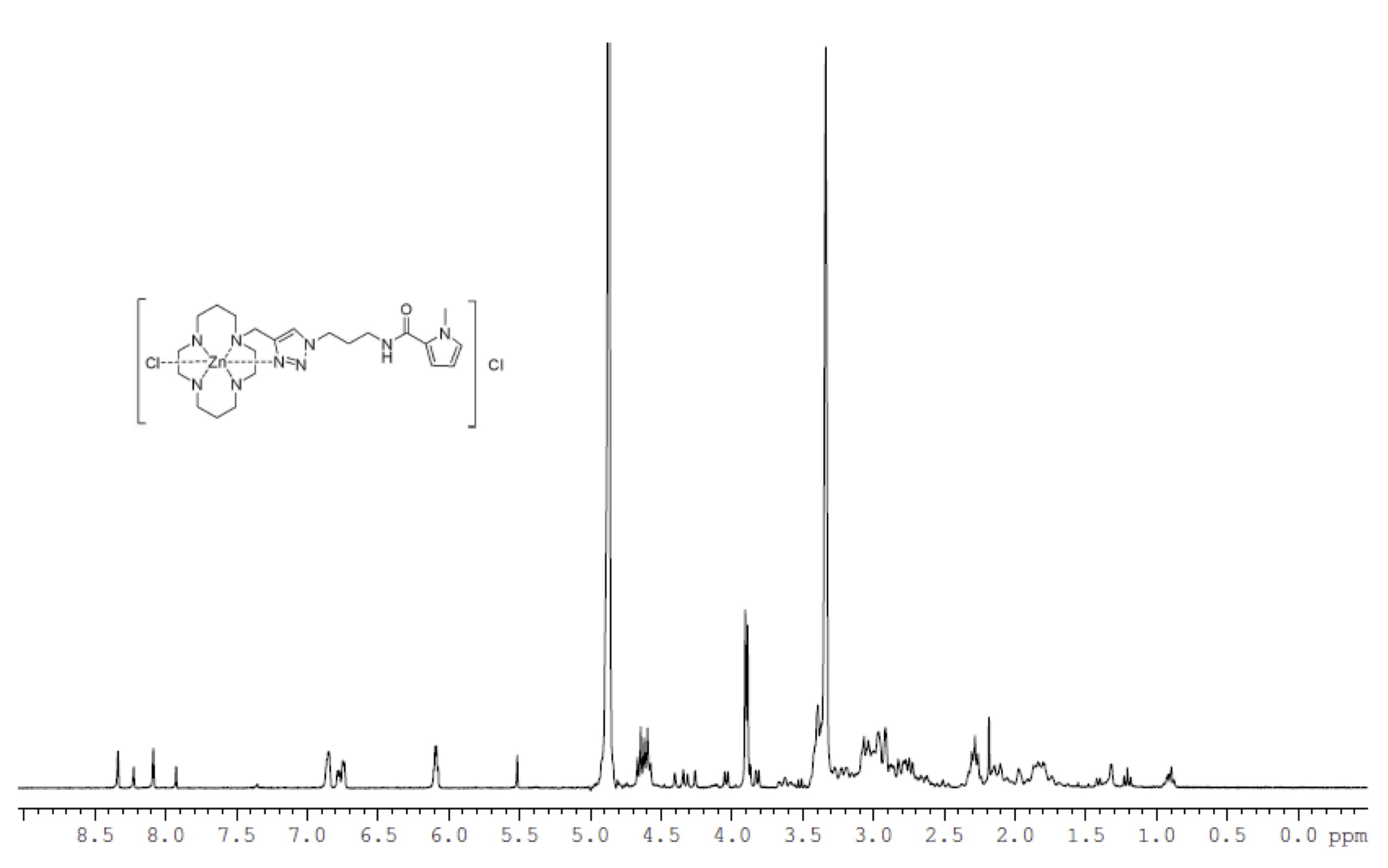

Supplement: Figure S19 — 300 MHz, MeOD, 1H NMR spectrum of mono-pyrrole zinc chloride cyclam complex (6a). (TIFF) [file pone.0017446.s020.tiff]

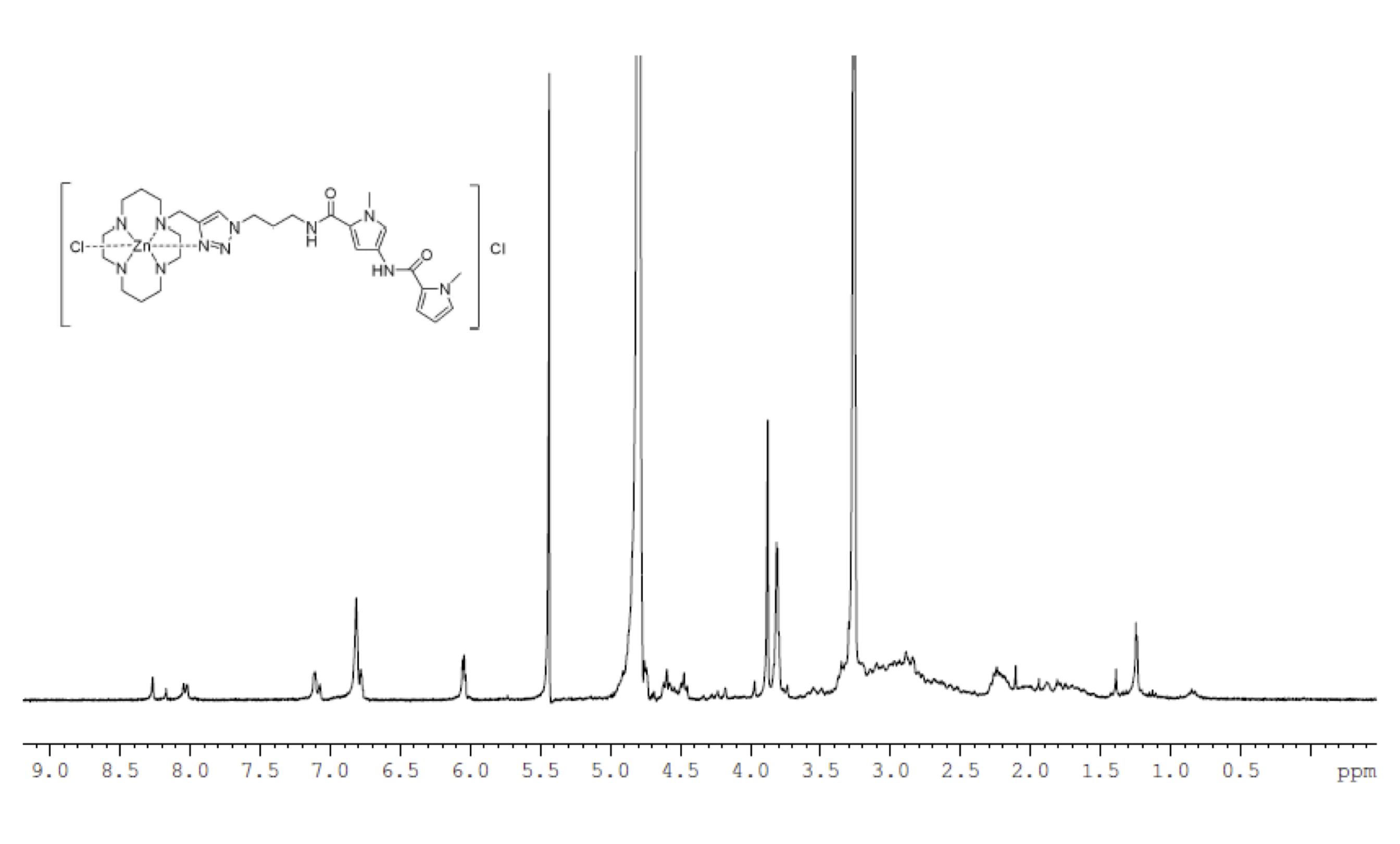

Supplement: Figure S20 — 300 MHz, MeOD, 1H NMR spectrum of di-pyrrole zinc chloride cyclam complex (6b). (TIFF) [file pone.0017446.s021.tiff]

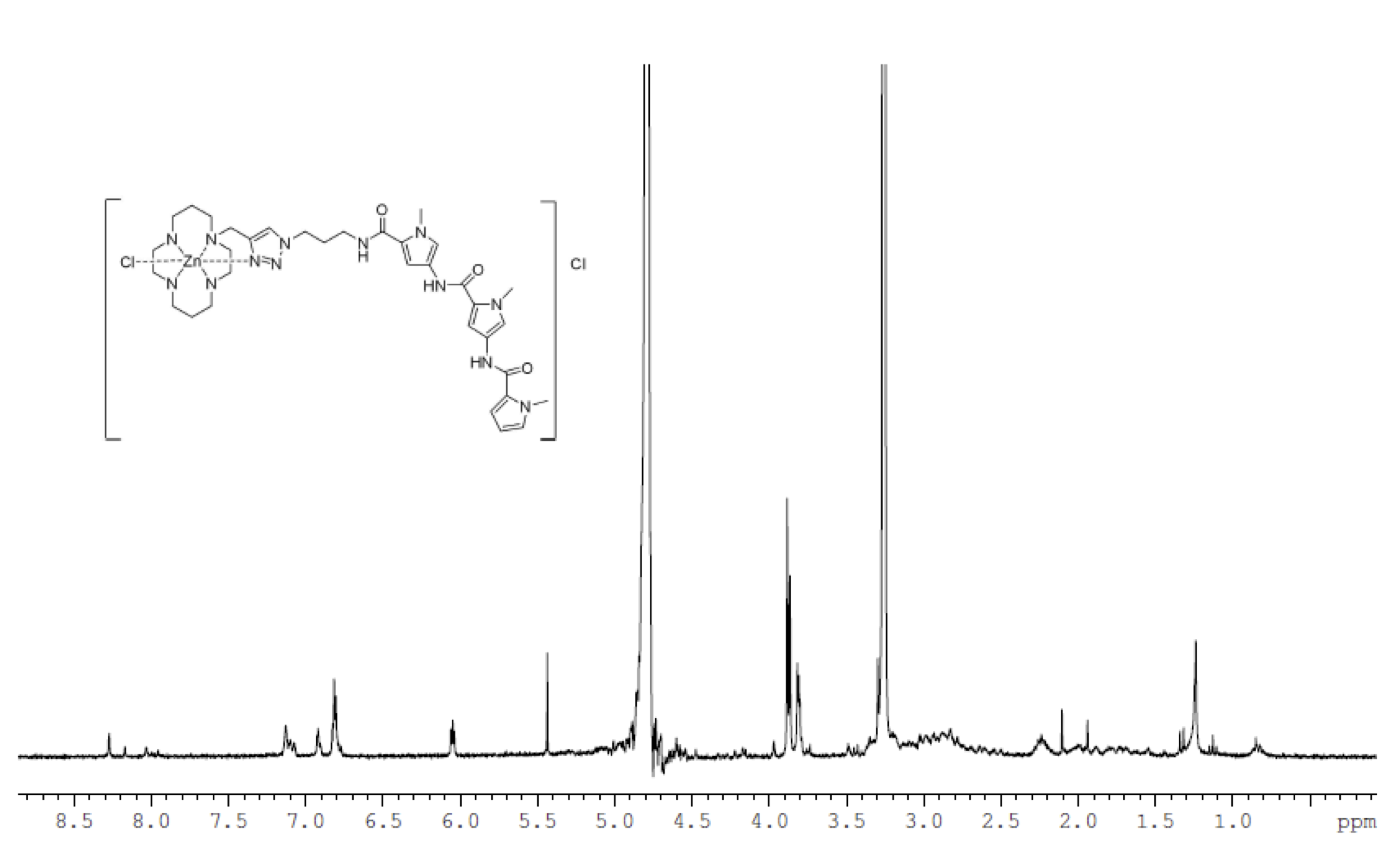

Supplement: Figure S21 — 300 MHz, MeOD, 1H NMR spectrum of tri-pyrrole zinc chloride cyclam complex (6c). (TIFF) [file pone.0017446.s022.tiff]

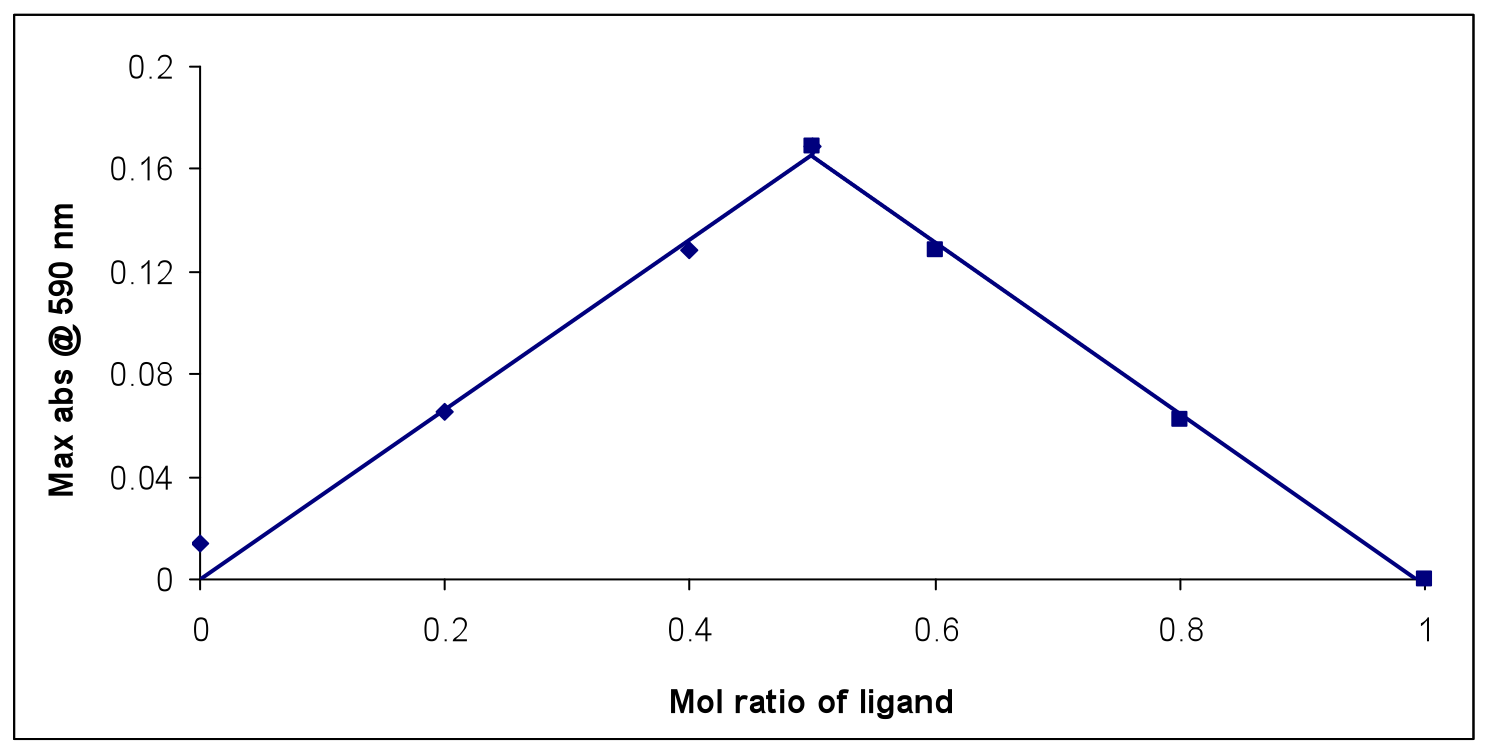

Supplement: Figure S22 — [125] Job plot for formation of complex between copper(II) and ligand 4a. (TIFF) [file pone.0017446.s023.tiff]

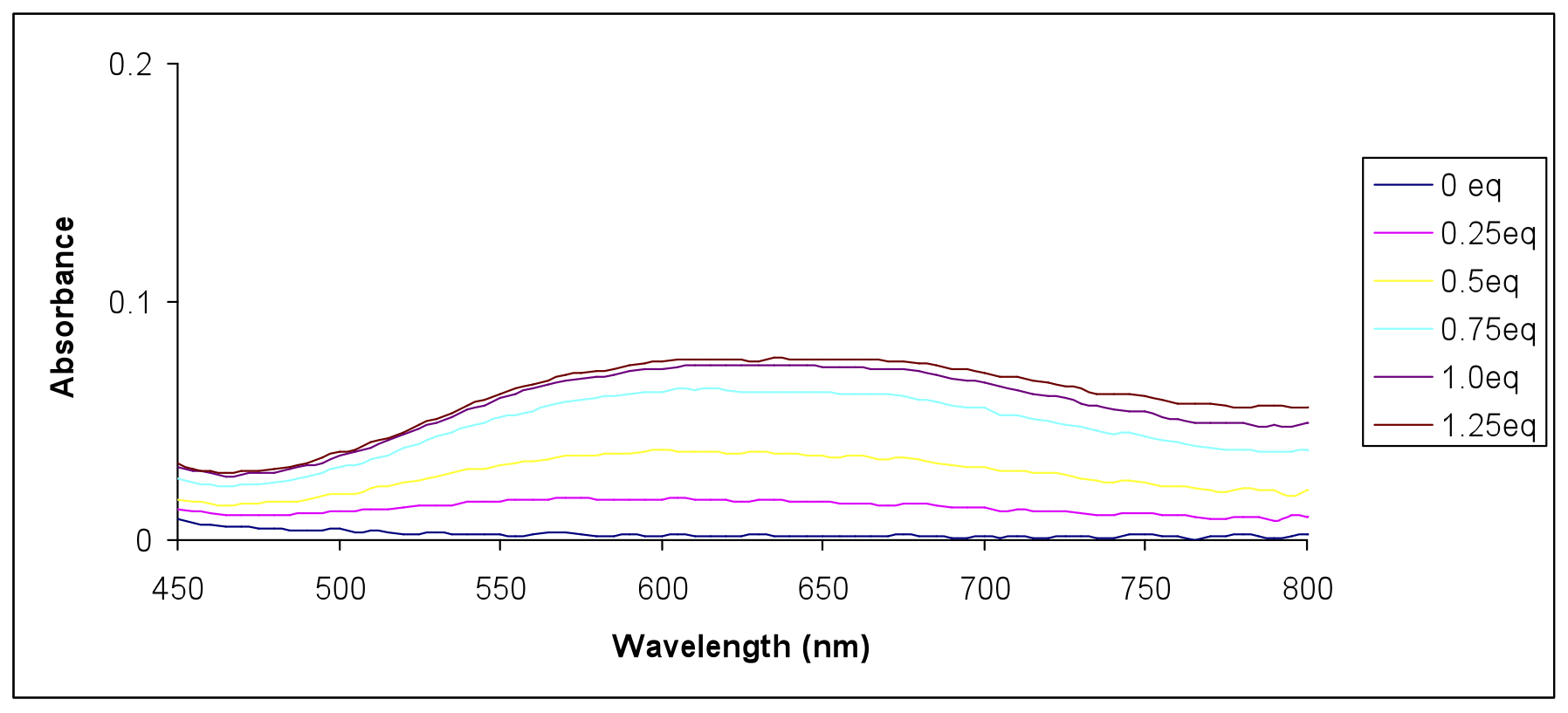

Supplement: Figure S23 — UV-vis spectrum for the titration of a solution of CuCl2 with compound 4c in methanol (graphical representation of raw data). (TIFF) [file pone.0017446.s024.tiff]

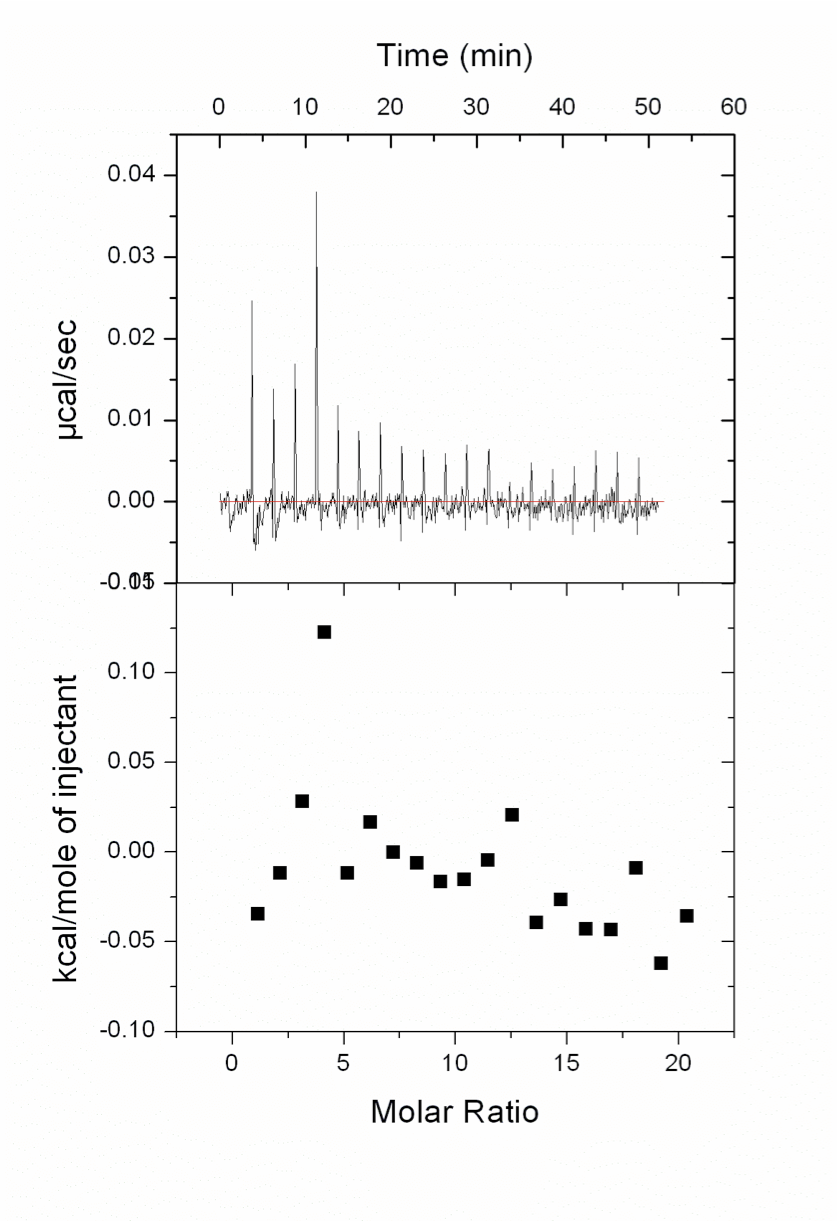

Supplement: Figure S24 — Example ITC curve for GC-rich oligonucleotide illustrating no observable binding; titration of 1000 µM 4c to 10 µM GC oligo (oligo II). (TIFF) [file pone.0017446.s025.tiff]
