# Supplementary material for: Polyamide-Scorpion Cyclam Lexitropsins Selectively Bind AT-Rich DNA Independently of the Nature of the Coordinated Metal
Source: PLoS One. 2011 May 9;6(5):e17446. doi: 10.1371/journal.pone.0017446 (PMC3090394; doi:10.1371/journal.pone.0017446)
Supplement: Text S1 — Procedures for preparation of known compounds, and description of entropy error calculations. (DOC) [file pone.0017446.s027.doc]

Supporting Information for

**Polyamide-scorpion Cyclam Lexitropsins Selectively Bind AT-rich DNA Independently of the Nature of the Coordinated Metal**

Anthony T. S. Lo,^§^ Noeris K. Salam^†^, David E. Hibbs^‡^, Peter J. Rutledge^§^ and Matthew H. Todd^§^*

^†^Schrödinger, Inc., 120 West 45th Street, 29th Floor, New York, New York 10036, USA.

^‡^Faculty of Pharmacy, University of Sydney, NSW 2006, Australia

^§^School of Chemistry, University of Sydney, NSW 2006, Australia

+61 2 9351 2180; matthew.todd@sydney.edu.au

**Contents**

Procedures for compound synthesis S3-S11

Method for calculation of error in ΔS S12

Supporting information references S12

Cyclam was synthesized according to literature methods.^S1^

1,4,8-Tris(*tert*-butoxycarbonyl)-1,4,8,11-tetraazacyclotetradecane **S1**^S2^

To cyclam (1.45 g, 7.23 mmol, 1 eq) in anhydrous dichloromethane (260 mL) under argon was added triethylamine (5.0 mL, 36 mmol, 5 eq) and stirring was continued for 15 min at rt. A solution of di-*tert*-butyl dicarbonate (4.75 g, 21.7 mmol, 3 eq) in anhydrous dichloromethane (90 mL) was dropwise over 3 h. Stirring was continued for 1 d. The solution was quenched with sodium carbonate (0.5 M, 400 mL) and the aqueous phase was extracted into dichloromethane (4 × 120 mL). The combined organic phases were dried (Na_2_SO_4_) and the solvent concentrated under reduced pressure yielding a pale yellow oil. Excess triethylamine was evaporated *in vacuo*. The residue was purified by flash column chromatography (ethyl acetate, *R*_F_ 0.10) yielding tri-Boc cyclam **S1** (2.68 g, 74%) as a white foam; **IR** (CHCl_3_) 1682, 1366, 1250 cm^-1^; **^1^H NMR** (CDCl_3_, 200 MHz) δ 3.37-3.27 (12H, m, H^a^), 2.78 (2H, t, *J* 5.2 Hz, H^b^), 2.61 (2H, t, *J* 5.4 Hz, H^c^), 2.00-1.80 (2H, m, H^d^), 1.80-1.60 (2H, m, H^e^), 1.46 (27H, s, C(CH_3_)_3_); **MS (ESI)** m/z 1023.7 (2M+Na^+^, 82%), 501.3 (MH^+^, 100%). Spectroscopic data matched those reported in the literature.^S2^

11-Prop-2-ynyl-1,4,8-tris(*tert*-butoxycarbonyl)-1,4,8,11-tetraazacyclotetradecane **S2** ^S2^

To protected cyclam **S1** (2.07 g, 4.13 mmol, 1 eq) in acetonitrile (110 mL) was added sodium carbonate (885.7 mg, 8.37 mmol, 2 eq) and propargyl bromide (80% wt. in toluene, 590.7 mg, 640 μL, 4.97 mmol, 1.2 eq) and the reaction mixture was heated at reflux at 82 °C for 2 d. The reaction mixture was filtered and the filtrate concentrated under reduced pressure and *in vacuo*. The residue was purified by flash column chromatography (7:3 ethyl acetate/hexane, *R*_F_ 0.73) to yield propargyl cyclam **S2** as white foam (1.66 g, 75%); **mp** 47-48 °C (lit.^S2^ 47-49 °C); **IR** (ATR) 2125, 1682, 1250, 1163 cm^-1^; **^1^H NMR** (CDCl_3_, 200 MHz) δ 3.45-3.20 (14H, m, H^a^), 2.67 (2H, t, *J* 4.6 Hz, H^b^), 2.51 (2H, t, *J* 3.5 Hz, H^c^), 2.16 (1H, s, CCH), 2.00-1.80 (2H, m, H^d^), 1.75-1.62 (2H, m, H^e^), 1.46 (27H, s, C(CH_3_)_3_); **MS (ESI)** m/z 1099.6 (2M+Na^+^, 100%), 561.5 (MNa^+^, 22%), 539.4 (MH^+^, 47%). Spectroscopic data matched those reported in the literature.^S2^

Tri-*tert*-butyl 11-((1-benzyl-1*H*-1,2,3-triazol-4-yl)methyl)-1,4,8,11-tetraazacyclotetradecane-1,4,8-tricarboxylate **S3**^S3^

The general ‘Click’ reaction Procedure B was followed using propargyl cyclam **S1** (1.40 g, 2.60 mmol, 0.93 eq) and benzyl azide (0.37 g, 2.79 mmol, 1 eq) to give a white solid, which was purified by flash column chromatography (ethyl acetate, *R*_F_ 0.50) yielding **S3** (1.42 g, 76%) as a white solid; **mp** 53-54 °C (lit.^S3^ 52-54 °C); **IR** (ATR) 2976, 1689 cm^-1^; **^1^H NMR** (CDCl_3_, 300 MHz) δ 7.30-7.40 (6H, m, Ar, H^g^), 5.50 (2H, s, PhC*H*_2_), 3.74 (2H, s, H^f^), 3.20-3.45 (12H, m, H^a^), 2.55-2.62 (2H, m, H^b^), 2.36-2.44 (2H, m, H^c^), 1.78-1.95 (2H, m, H^d^), 1.63-1.78 (2H, m, H^e^), 1.43 (27H, s, C(CH_3_)_3_); **^13^C NMR** (CDCl_3_, 50.3 MHz) δ 155.2, 154.9, 134.5, 128.6, 128.1, 127.4, 122.1, 78.9, 53.5, 47.0-45.5 (multiple peaks), 45.0; **MS (ESI)** m/z 672.1 (MH^+^, 67%), 694.3 (MNa^+^, 100%); **HRMS (ESI)** calcd. for C_35_H_58_N_7_O_6_^+^ 672.44486 found 672.44431 (MH^+^). Spectroscopic data matched those reported in literature.^S3^

1-((1-Benzyl-1-H-1,2,3-triazol-4-yl)methyl)-1,4,8,11-tetraazacyclotetradecane **S4** ^S3^

The general TFA deprotection Procedure C was followed using benzyl tri-Boc cyclam **S3** (1.01 g, 1.50 mmol, 1 eq) yielding **S4** (0.55 g, 99%) as a white solid; **mp** 105-106 °C; **IR** (ATR) 3269, 2812, 1458 cm^-1^; **^1^H NMR** (CDCl_3_, 300 MHz) δ 7.45 (1H, s, H^g^), 7.27-7.42 (5H, m, Ar), 5.50 (2H, s, H^h^), 3.75 (2H, s, H^f^), 2.48-2.70 (12H, m, H^a^), 1.77-1.85 (2H, m, H^d^), 1.62-1.76 (3H, br s, NH), 1.53-1.62 (2H, m, H^e^); **^13^C NMR** (CDCl_3_, 75.5 MHz) δ 144.0 (Ar), 133.8 (Ar), 127.7 (2Ar), 127.1 (Ar), 127.0 (2Ar), 121.0 (Ar), 53.5, 52.5, 52.2, 49.6, 48.6, 47.9, 47.4, 46.8, 46.6, 46.0, 27.6, 24.9; **MS (ESI)** m/z 372.3 (MH^+^, 100%); **HRMS (ESI)** calcd. for C_20_H_34_N_7_^+^ 372.28757 found 372.28702 (MH^+^).

2-Trichloroacetyl-1-methylpyrrole **S5**^S4^

A solution of 1-methylpyrrole (11.0 mL, 123 mmol, 1 eq) in anhydrous dichloromethane (40 mL) was added dropwise to a solution of trichloroacetyl chloride (15.2 mL, 123 mmol, 1 eq) in anhydrous dichloromethane (40 mL) cooled to 0 °C. The reaction was stirred for 16 h. The solvent was removed under reduced pressure and the residue was purified by flash column chromatography (3:7 dichloromethane/hexane, *R*_F_ 0.30) to yield **S5** (24.5 g, 88%) as a pale yellow solid. Alternatively, recrystallisation from methanol gave the product as white needles (5.62 g, 29%); **mp** 64-65 °C (lit.^S4^ 65-66 °C); **IR** (CHCl_3_) 1666, 1516, 1211, 717 cm^-1^; **^1^H NMR** (CDCl_3_, 200 MHz) δ 7.50 (1H, dd, *J* 4.4, 1.5 Hz, Ar), 6.98 (1H, d, *J* 1.5 Hz, Ar), 6.22 (1H, dd, *J* 4.4 & 2.4 Hz, Ar), 3.98 (3H, s, CH_3_); **MS (ESI)** m/z 292.5 (100%). Spectroscopic data matched those reported in the literature. ^S4^

4-Bromo-2-trichloroacetyl-1-methylpyrrole **S6** ^S4^

*N*-Bromosuccinimide (3.50 g, 19.7 mmol, 1 eq) was added to 2-trichloroacetyl-1-methylpyrrole **S5** (4.45 g, 19.6 mmol, 1 eq) in anhydrous chloroform (55 mL) under nitrogen and cooled to -5 °C. Stirring was continued at 0 °C for 2 h and then at rt for 16 h. The solvent was removed under reduced pressure and the residue was purified by flash column chromatography (1:9 dichloromethane/hexane, *R*_F_ 0.28), to obtain the bromomethylpyrrole **S6** as a white solid (5.57 g, 93%). Alternatively, recrystallisation of the crude residue from ethanol gave the bromomethylpyrrole as white needles (4.13 g, 69%); **mp** 102-104 °C (lit.^S4^ 105-107 °C); **IR** (CHCl_3_) 1682, 1520, 1366, 717, 656 cm^-1^; **^1^H NMR** (CDCl_3_, 200 MHz) δ 7.46 (1H, d, *J* 1.6 Hz, Ar), 6.96 (1H, d, *J* 1.6 Hz, Ar), 3.95 (3H, s, CH_3_); **MS (EI)** m/z 301.9 (MH^+^, multiplet, 10%), 188.0 (C_6_H_5_O^81^Br^+^, 90%), 186.0 (C_6_H_5_O^79^Br^+^, 100%). Spectroscopic data matched those reported in the literature. ^S4^

Methyl 4-bromomethylpyrrole-2-carboxylate **S7** ^S4^

4-Bromo-2-trichloroacetyl-1-methylpyrrole **S6** (50.3 mg, 0.16 mmol, 1 eq) was dissolved in anhydrous methanol (1.5 mL). Sodium methoxide (25 wt% in methanol, 45 μL, 0.20 mmol, 1.2 eq) was added dropwise and the reaction mixture was stirred for 2 h. The reaction was cooled to 0 °C and quenched with hydrochloric acid (0.02 M, 2 mL) dropwise. The solvent was concentrated under reduced pressure and water (4 mL) and dichloromethane (10 mL) were added. The aqueous phase was extracted with dichloromethane (2 × 10 mL), and the combined organic phases dried (Na_2_SO_4_) and concentrated yielding **S7** (27.3 mg, 84%) as white needles; **mp** 64-65 °C (lit. ^S4^ 65-66 °C); **IR** (CHCl_3_) 1713, 1520, 1396, 1258, 671 cm^-1^; **^1^H NMR** (CDCl_3_, 200 MHz) δ 6.91 (1H, d, *J* 2.0 Hz, Ar), 6.77 (1H, d, *J* 2.0 Hz, Ar), 3.90 (3H, s, NCH_3_), 3.81 (3H, s, OCH_3_); **MS (ESI)** m/z 219.1 (MH^+^, 10%), 218.1 (C_6_H_6_NO_2_^81^Br^+^, 50%), 216.1 (C_6_H_6_NO_2_^79^Br^+^, 50%), 187.8 (C_5_H_3_NO^81^Br^+^, 64%), 185.9 (C_5_H_3_NO^79^Br^+^, 68%), 81.5 (C_5_H_5_N^+^, 100%). Spectroscopic data matched those reported in the literature. ^S4^

Methyl 4-[(*tert*-butoxycarbonyl)amino]-1-methylpyrrole-2-carboxylate **S8** ^S4^

To ground potassium phosphate (2.28 g, 10.7 mmol, 2 eq) in 1,4-dioxane (30 mL) were added copper(I) iodide (205 mg, 1.07 mmol, 0.2 eq) and *N*,*N*’-dimethylethylenediamine (231 μL, 2.1 mmol, 0.4 eq) and the reaction mixture was stirred under argon. Methyl 4-bromomethylpyrrole-2-carboxylate **S7** (1.17 g, 5.4 mmol, 1 eq) and *tert*-butyl carbamate (0.65 g, 5.50 mmol, 1.03 eq) were added. The mixture was heated at reflux for 72 h, diluted with ethyl acetate and filtered through a pad of Celite. The solvent was concentrated under reduced pressure to yield a white solid, which was purified by flash column chromatography (1:49 ethyl acetate/hexane, R_f_ 0.10) to give **S8** as a white solid (1.20 g, 88%); **mp** 113-114 °C (lit. ^S4^ 115-116 °C); **IR** (CHCl_3_) 3448, 1713, 1520, 1389, 1265 cm^-1^; **^1^H NMR** (CDCl_3_, 200 MHz) δ 7.09 (1H, s, Ar), 6.61 (1H, s, Ar), 6.25 (1H, s, NH), 3.86 (3H, s, NCH_3_), 3.78 (3H, s, OCH_3_), 1.49 (9H, s, C(CH_3_)_3_); **MS (ESI)** m/z 254.8 (MH^+^, 78%) Spectroscopic data matched those reported in the literature. ^S4^

4-[(*tert*-Butoxycarbonyl)amino]-1-methylpyrrole-2-carboxylic acid **S9** ^S4^

The general ester deprotection Procedure D was followed using methyl 4-[(*tert*-butoxycarbonyl)amino]-1-methylpyrrole-2-carboxylate **S8** (650 mg, 2.56 mmol, 1 eq). The residue was purified by flash column chromatography (1:1 ethyl acetate/hexane, *R*_F_ 0.26) to yield a pale yellow solid, which was recrystallised by ethyl acetate and the addition of cold hexane to yield **S9** (0.45 g, 74%) as white crystals; **mp** 159-160 °C (lit. ^S4^ 160 °C); **IR** (CHCl_3_) 3448, 3200-2800, 1713, 1520, 1389, 1227 cm^-1^; **^1^H NMR** (CDCl_3_, 200 MHz) δ 7.17 (1H, br s, NH), 6.71 (1H, d, *J* 2.0 Hz, Ar), 6.20-6.28 (1H, m, Ar), 3.87 (3H, s, NCH_3_), 1.50 (9H, s, C(CH_3_)_3_); **MS (ESI)** m/z 239.3 (C_11_H_15_N_2_O_4_^+^, 100%). Spectroscopic data matched those reported in literature. ^S4^

Methyl 4-amino-1-methyl-1*H*-pyrrole-2-carboxylate **S10**^S2^

The general Boc deprotection Procedure C was followed using methyl 4-[(*tert*-butoxycarbonyl)amino]-1-methylpyrrole-2-carboxylate **S8** (219 mg, 0.58 mmol, 1 eq) to give the amine **S10** (0.16 g, 100%) as a light yellow oil; **IR** (ATR) 3395, 3347, 1692, 1577 cm^-1^; **^1^H NMR** (CDCl_3_, 200 MHz) δ 6.46 (1H, d, *J* 2.2 Hz, Ar), 6.36 (1H, d, *J* 2.2 Hz, Ar), 3.81 (3H, s, N(CH_3_)), 3.77 (3H, s, N(CH_3_)), 3.75 (2H, br s, NH_2_); **^13^C NMR** (CDCl_3_, 75.5 MHz) δ 162.1 (C=O), 130.7 (Ar), 120.7 (Ar), 118.2 (Ar), 108.7 (Ar), 51.3, 36.7; **MS** (ESI) m/z 155.2 (C_7_H_11_N_2_O_2_^+^, 47%), 440.4 (100%). This compound decomposes quickly; satisfactory HRMS could not be obtained.

Methyl 1-methylpyrrole-2-carboxylate **S11**^S5^

1-Methylpyrrole (3.1 mL, 34.8 mmol, 1 eq) was stirred in anhydrous tetrahydrofuran (120 mL) under argon. The reaction mixture was cooled to -78 °C and *n*-butyllithium (1.8 M in hexanes, 22 mL, 39.5 mmol, 1.1 eq) was added dropwise over 3 h. Stirring was continued for 30 min at -78 °C and then for 1 h at 0 °C. The reaction mixture was cooled -78 °C and methyl chloroformate (3.0 mL, 38.7 mmol, 1 eq) was added dropwise to the reaction mixture and stirring was continued for 1 h. The reaction was warmed to room temperature and stirring was continued for 16 h. Water (20 mL) was added. The tetrahydrofuran was removed under reduced pressure and the residue was extracted with dichloromethane (3 × 50 mL) to yield a yellow oil, which was purified by column flash chromatography (1:1 ethyl acetate/hexane, *R*_F_ 0.51); yielding **S11** (1.38 g) as a pale yellow oil, which was used directly in the next reaction without purification.

1-Methylpyrrole-2-carboxylic acid **1a**^S6^

The general ester deprotection Procedure D was followed using methyl carboxylate **S11** (1.38 g, 9.91 mmol, 1 eq) to give the pyrrole carboxylic acid **1a** (0.58 g, 46%) over two steps as white crystals (1:1 ethyl acetate/hexane, *R*_F_ 0.19); **mp** 133-134 °C (lit.^S7^ 135-136 °C); **IR** (ATR) 2953, 1669 cm^-1^; **^1^H NMR** (CDCl_3_, 200 MHz) δ 7.06-7.10 (1H, m, Ar), 6.82-6.86 (1H, m, Ar), 6.12-6.17 (1H, m, Ar), 3.92 (3H, s, CH_3_), 1.38 (1H, br s, OH). MS (ESI) m/z 126.1 (C_6_H_8_NO_2_^+^, 30%) Spectroscopic data matched those reported in the literature.^S7^

Methyl 1-methyl-4-(1-methyl-1*H*-pyrrole-2-carboxamido)-1*H*-pyrrole-2-carboxylate **S12**^S8^

The general coupling Procedure A was followed using methyl ester protected methylpyrrole amine **S10** (192 mg, 1.25 mmol, 1.3 eq) and the methylpyrrole carboxylic acid **1a** (120 mg, 0.96 mmol, 1 eq). The residue was purified by flash column chromatography (1:1 ethyl acetate/hexane, *R*_F_ 0.46); yielding **S12** (138 mg, 55%) as a white solid; **mp** 102 °C (lit.^S9^ 102 °C); **IR** (ATR) 3337, 2949, 1703 cm^-1^; **^1^H NMR** (CDCl_3_, 300 MHz) δ 7.44 (1H, br s, NH), 7.42 (1H, d, *J* 1.9 Hz, Ar), 6.73-6.78 (2H, m, 2 Ar), 6.62 (1H, dd, *J* 3.9 & 1.6 Hz, Ar), 6.12 (1H, dd, *J* 3.9 & 2.6 Hz, Ar), 3.97 (3H, s, NCH_3_), 3.90 (2H, s, NCH_3_), 3.80 (3H, s, OCH_3_); **MS** (ESI) m/z 262.1 (MH^+^, 100%). Spectroscopic data matched those reported in the literature.^S9^

1-Methyl-4-(1-methyl-1*H*-pyrrole-2-carboxamido)-1*H*-pyrrole-2-carboxylic acid **2a**^S8^

The general coupling Procedure A was followed using methyl ester protected pyrrole amide **S12** (135 mg, 0.52 mmol, 1 eq) to give the methylpyrrole amide carboxylic acid **2a** (71 mg, 55%) as a white solid. **mp** 174-175 °C (lit.^S9^ 175 °C); **IR** (ATR) 3560, 1670 cm^-1^; **^1^H NMR** (MeOD, 300 MHz) δ 7.33 (1H, d, *J* 1.9 Hz, Ar), 6.96 (1H, d, *J* 1.9 Hz, Ar), 6.87 (1H, dd, J 4.0 & 1.7 Hz, Ar), 6.83-6.86 (1H, m, Ar), 6.09 (1H, *J* 4.0 & 2.6 Hz, Ar), 3.93 (3H, s, N(CH_3_)), 3.90 (3H, s, N(CH_3_)); **^13^C NMR** (MeOD, 75.5 MHz) δ 164.2 (C=O), 161.7 (C=O), 129.6 (Ar), 126.7 (Ar), 123.7 (Ar), 122.4 (Ar), 121.4 (Ar), 114.2 (Ar), 110.8 (Ar), 108.3 (Ar), 37.0, 36.9; **MS (ESI)** m/z 248.1 (MH^+^, 38%); **HRMS** (ESI) calcd for C_12_H_13_N_3_NaO_3_^+^ 270.08546 (MNa^+^) found 270.08491. Spectroscopic data matched those reported in the literature.^S9^

Methyl 4-(4-(*tert*-butoxycarbonylamino)-1-methyl-1*H*-pyrrole-2-carboxamido)-1-methyl-1*H*-pyrrole-2-carboxylate **S13**^S8^

The general coupling Procedure A was followed using methyl ester protected methylpyrrole amine **S10** (208 mg, 1.35 mmol, 1.3 eq) and the Boc-protected methylpyrrole carboxylic acid **S9** (250 mg, 1.04 mmol, 1 eq). The residue was purified by flash column chromatography (ethyl acetate, *R*_F_ 0.56) to give **S13** as a light yellow solid (222 g, 44%); **mp** 88-90 °C; **IR** (ATR) 3379, 1707 cm^-1^; **^1^H NMR** (CDCl_3_, 200 MHz) δ 7.39-7.45 (2H, m, Ar, NH), 6.80-6.85 (1H, m, Ar), 6.72-6.75 (1H, m, Ar), 6.55-6.60 (1H, m, Ar), 6.16-6.26 (1H, m, Ar), 3.90 (6H, s, 2 N(CH_3_)), 3.81 (3H, s, OCH_3_), 1.51 (9H, s, C(CH_3_)_3_); **^13^C NMR** (CDCl_3_, 50.3 MHz) δ 161.5 (C=O), 158.9 (C=O), 153.5 (C=O), 123.2 (Ar), 121.8 (Ar), 121.7 (Ar), 120.9 (Ar), 119.8 (Ar), 118.4 (Ar), 108.2 (Ar), 103.6 (Ar), 80.3 (*C*(CH_3_)_3_), 51.1 (OCH_3_), 36.7 (NCH_3_), 36.5 (NCH_3_); **MS (ESI)** m/z 377.1 (MH^+^, 22%), 399.1 (MNa^+^, 100%), **HRMS (ESI)** calcd for C_18_H_25_N_4_O_5_^+^ 377.18249 found 377.18195 (MH^+^). Spectroscopic data matched those reported in the literature.^S10^

Methyl 4-(4-amino-1-methyl-1*H*-pyrrole-2-carboxamido)-1-methyl-1*H*-pyrrole-2-carboxylate **S14**^S2^

The general Boc deprotection Procedure C was followed using the Boc and methyl ester protected pyrrole amide **S13** (219 mg, 0.58 mmol, 1 eq) to give the amine **S14** (159 mg, 99%) as a light yellow gum; **IR** (ATR) 3326, 1700 cm^-1^; **^1^H NMR** (CDCl_3_, 300 MHz) δ 7.40 (1H, d, *J* 1.9 Hz, Ar), 7.34 (1H, br s, NH), 6.72 (1H, d, *J* 2.0 Hz, Ar), 6.33 (1H, d, *J* 1.9 Hz, Ar), 6.18 (1H, d, *J* 2.0 Hz, Ar), 2.00 (2H, br s, NH); **MS** (ESI) m/z 277.0 (MH^+^, 92%). Spectroscopic data matched those reported in the literature.^S10^

Methyl 1-methyl-4-(1-methyl-4-(1-methyl-1*H*-pyrrole-2-carboxamido)-1*H*-pyrrole-2-carboxamido)-1*H*-pyrrole-2-carboxylate **S15**^S8^

The general coupling Procedure A was followed using methyl ester protected methylpyrrole amide amine **S14** (143 mg, 0.52 mmol, 1.1 eq) and methylpyrrole carboxylic acid **1a** (59 mg, 0.47 mmol, 1 eq). The residue was purified by flash column chromatography (ethyl acetate, *R*_F_ 0.60) giving **S15** (102 mg, 57%) as a white solid; **mp** 134-135 °C (lit.^S9^ 135 °C) ; **IR** (ATR) 3337, 1704 cm^-1^; **^1^H NMR** (CDCl_3_, 300 MHz) δ 7.41-7.48 (4H, 2 NH & 2 Ar), 7.13-7.16 (1H, m, Ar), 6.76-6.69 (1H, m, Ar), 6.72-6.75 (1H, m, Ar), 6.62-6.66 (1H, m, Ar), 6.13-6.17 (1H, m, Ar), 3.98 (3H, s, NCH_3_), 3.95 (3H, s, NCH_3_), 3.91 (3H, s, NCH_3_), 3.81 (3H, s, OCH_3_); **MS** (ESI) m/z 384.1 (MH^+^, 70%), 230.1 (100%). Spectroscopic data matched those reported in the literature.^S9^

1-Methyl-4-(1-methyl-4-(1-methyl-1*H*-pyrrole-2-carboxamido)-1*H*-pyrrole-2-carboxamido)-1*H*-pyrrole-2-carboxylic acid **3a**^S9^

The general ester deprotection Procedure D was followed using methyl ester protected methylpyrrole amide **S15** (100 mg, 0.26 mmol, 1 eq) to give the methylpyrrole amide carboxylic acid **3a** (60 mg, 62%) as a white solid; **mp** 174-175 °C (lit.^S9^ 175 °C); **IR** (ATR) 3439, 1717, 1684 cm^-1^; **^1^H NMR** (DMSO-d_6_, 200 MHz) δ 9.87 (1H, br s, NH), 9.81 (1H, br s, NH), 7.38-7.43 (1H, m, Ar), 7.22-7.25 (1H, m, Ar), 7.01-7.08 (1H, m, Ar), 6.88-7.00 (2H, m, 2 Ar), 6.80-6.89 (1H, m, Ar), 6.03-6.11 (1H, m, Ar), 3.88 (3H, s, NCH_3_), 3.84 (3H, s, NCH_3_), 3.82 (3H, s, NCH_3_), 2.50 (1H, br s, COOH); **MS** (ESI) m/z 370.1 (MH^+^, 44%), 230.1 (100%). Spectroscopic data matched those reported in the literature.^S9^

***Synthesis of Non-lexitropsin Cyclam Complexes***

[Cu-cyclam]Cl_2_. Copper(II) chloride was inserted into cyclam (2.88 mg, 14.4 µmol, 1.0 eq) according to general procedure D. The solution was made up to 3 mL in methanol in the 3 mL cuvette to a final concentration of 4.8 mM; **UV-vis** (MeOH) λ_max_ = 528 nm, ε = 83 M^-1^ cm^-1^; **IR** (ATR) 3410, 2943, 2876, 1653, 1432, 1205, 1102, 887 cm^-1^; **MS** (ESI) m/z 297.9 (C_11_H_27_N_4_OCu^+^, 100%), 299.9 (C_11_H_27_^35^ClN_4_OCu^+^, 80%).^S13^

[Zn-cyclam]Cl_2_. Zinc(II) chloride was inserted into cyclam (2.9 mg, 14.5 µmol, 1.0 eq) according to general procedure D. **^1^H NMR** (D_2_O) proton peaks were unable to be assigned but corresponded to literature.^S12^

[Cu(**S4**)Cl]Cl. Copper(II) chloride was inserted into benzyl cyclam ligand **S4** (2.8 mg, 7.6 μmol, 1.0 eq) according to general procedure D. The solution was made up to 3 mL in methanol to a final concentration of 2.5 mM; **UV-vis** (MeOH) λ_max_ = 585 nm, ε = 135 M^-1^ cm^-1^; **MS** (ESI) m/z 471.0 (C_20_H_33_^35^ClCuN_7_^+^, 58%).^S3^

[Zn(**S4**)Cl]Cl. Zinc(II) chloride was inserted into benzyl cyclam ligand **S4** (6.6 mg, 14 μmol, 1.0 eq) according to general procedure D. **MS** (ESI) m/z 473.1 (C_20_H_33_N_7_^35^ClZn^+^, 100%).^S3^

**Method for calculation of error in ΔS**

From ΔG = ΔH – TΔS and ΔG = -RTlnK, ΔS = ΔH/T + RlnK

When adding quantities, error propagation gives composite error as the root of the constituent errors squared, hence:

ΔS_error_ = Sqrt[(R.K_error_/K)^2 + (ΔH_error_/T)^2]

assuming that error in T can be neglected. This also uses: error in f(x) = x_error_. d(f(x))/dx, hence error in lnK = K_error_.d(lnK)/dK = K_error_/K

Values are calculated in the spreadsheet available as supporting information.

**References**

S1. Barefield EK, Wagner F, Herlinger AW, Dahl AR, Holt S (2007) (1,4,8,11-Tetraazacyclotetradecane)Nickel(II) perchlorate and 1,4,8,11-tetraazacyclotetradecane. Inorg Syn 16: 220-225. (10.1002/9780470132470).

S2. Tamanini E, Katewa A, Sedger LM, Todd MH, Watkinson M (2009) A synthetically simple, click-generated cyclam-based zinc(II) sensor. Inorg Chem 48: 319-324. (10.1021/ic8017634)

S3. Tamanini E, Rigby SEJ, Motevalli M, Todd MH, Watkinson M (2009) Responsive metal complexes: a click-based "allosteric scorpionate" complex permits the detection of a biological recognition event by EPR/ENDOR spectroscopy. Chem Eur J **15**: 3720-3728. (10.1002/chem.200802425)

S4. Jaramillo D, Liu Q, Aldrich-Wright J, Tor Y (2004) Synthesis of N-methylpyrrole and N-methylimidazole amino acids suitable for solid-phase synthesis. J Org Chem 69: 8151-8153. (10.1021/jo048686r)

S5. Bakhtiar C, Smith EH (1994) Transfer of alkoxycarbonyl from alkyl imidazolium-2-carboxylates to benzyl alcohol, a cyclohexanone enamine and diethylamine. J Chem Soc-Perkin Trans I 239-243. (10.1039/P19940000239)

S6. Teng X, Keys H, Yuan J, Degterev A, Cuny GD (2008) Structure-activity relationship and liver microsome stability studies of pyrrole necroptosis inhibitors. Bioorg Med Chem Lett 18: 3219-3223. (10.1016/j.bmcl.2008.04.048)

S7. Shirley DA, Gross BH, Roussel PA (1955) Metalation of pyrrole, 1-methylpyrrole, and 1-phenylpyrrole with normal-butyllithium J Org Chem 20: 225-231. (10.1021/jo01120a012)

S8. Cuenca F, Moore MJB, Johnson K, Guyen B, De Cian A, Neidle S (2009) Design, synthesis and evaluation of 4,5-disubstituted acridone ligands with high G-quadruplex affinity and selectivity, together with low toxicity to normal cells. Bioorg Med Chem Lett 19: 5109-5113. (10.1016/j.bmcl.2009.07.033)

S9. Thomas M, Varshney U, Bhattacharya S (2002) Distamycin analogues without leading amide at their N-termini - comparative binding properties to A•T- and G•C-rich DNA sequences. Eur J Org Chem 2002: 3604-3615. (10.1002/1099-0690(200211)2002:21<3604::AID-EJOC3604>3.0.CO;2-X)

S10. Chenoweth DM, Harki DA, Dervan PB (2009) Solution-phase synthesis of pyrrole-imidazole polyamides. J Am Chem Soc 131: 7175-7181. (10.1021/ja901307m)

S11. De Buysser K, Herman GG, Bruneel E, Hoste S, Van Driessche I (2005) Determination of the number of unpaired electrons in metal-complexes. A comparison between the Evan’s method and susceptometer results, Chem Phys 315: 286-292. (10.1016/j.chemphys.2005.04.022)

S12. Liang X, Weishäupl M, Parkinson JA, Parsons S, McGregor PA, Sadler PJ (2003) Selective recognition of configurational subtrates of zinc cyclam by carboxylates: implications for the design and mechanism of action of anti-HIV agents. Chem Eur J 9: 4709-4717. (10.1002/chem.200304808)
