# Supplementary material for: Polyamide-Scorpion Cyclam Lexitropsins Selectively Bind AT-Rich DNA Independently of the Nature of the Coordinated Metal
Source: PLoS One. 2011 May 9;6(5):e17446. doi: 10.1371/journal.pone.0017446 (PMC3090394; doi:10.1371/journal.pone.0017446)
Supplement: Table S1 — Effect of structural modifications of lexitropsins on binding affinities for compound 4c vs. selected literature compounds. (DOC) [file pone.0017446.s028.doc]

**Supplemental Table**. Effect of Structural Modifications of Lexitropsins on Binding affinities K_a_ (× 10^5^ M^-1^) for compound **4c** (Entry 7) *vs*. selected literature compounds. Figures in bold are for cognate sequences, regular text are for unoptimised or alien sequences (shown). Conditions: A: SPR-based assay, 10 mM MES (2-(*N*-morpholino)ethanesulfonic acid), pH 6.2, buffer with 200 mM NaCl, 1 mM EDTA (disodium ethylenediamine tetraacetic acid), and 0.001% P20 surfactant, 25 °C. B: SPR-based assay, degassed phosphate buffer (200 mM Na^+^, 10 mM sodium phosphate, 1 mM EDTA, and 0.00005 v/v of 10% surfactant P20, pH 6.25), 25 °C. C: 10 mM HEPES, 100 mM sodium chloride, pH 7.0; ^a^For ATATAT.

| Entry | Lexitropsin | K_a_ for AAATTT | Comparison Sequence | K_a_ for Comparison Sequence | Condns. | Ref |
| --- | --- | --- | --- | --- | --- | --- |
| 1 | 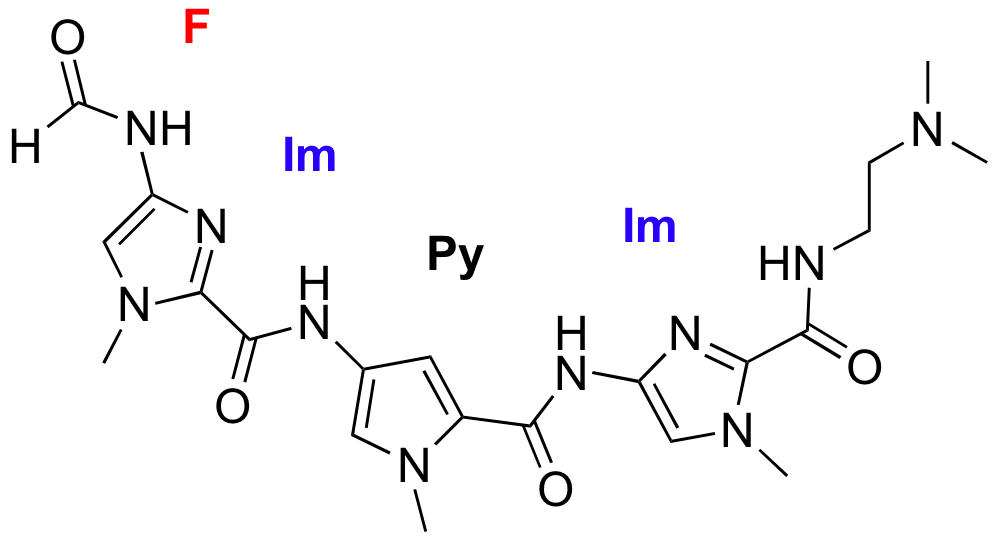 |  | ACGCGT | **1900** | A | 46 |
| 2 | 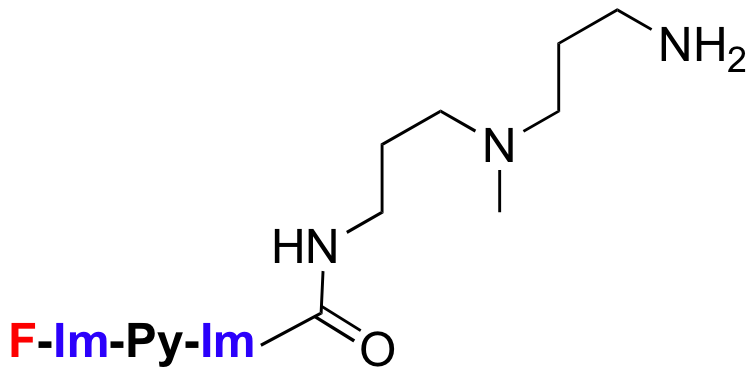 | 1 |  | **22** | B |  |
| 3 | 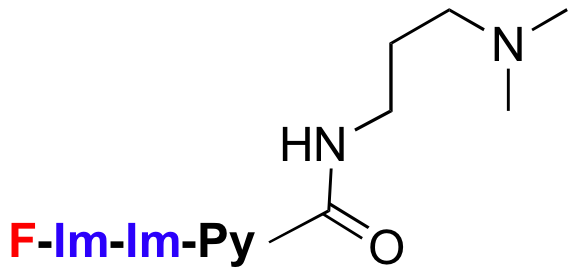 | 0.58 | ATGCAT | **1.1** | A | 8f |
| 4 | 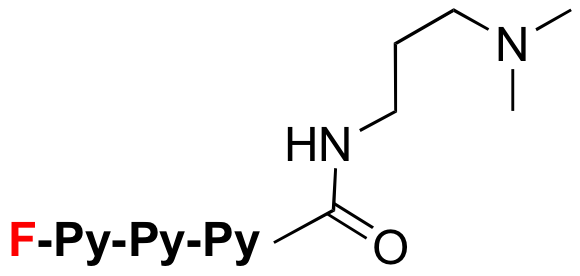 | **32** |  |  |  |  |
| 5 | 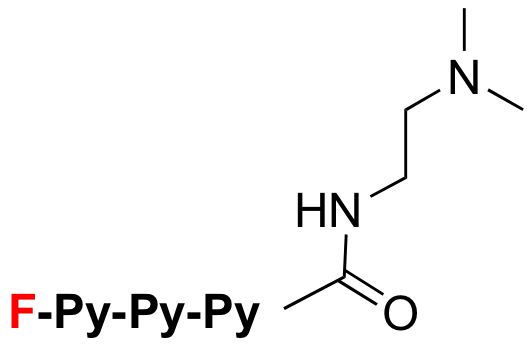 | **32** | ACTGGT | 0.79 | A | 39 |
| 6 | 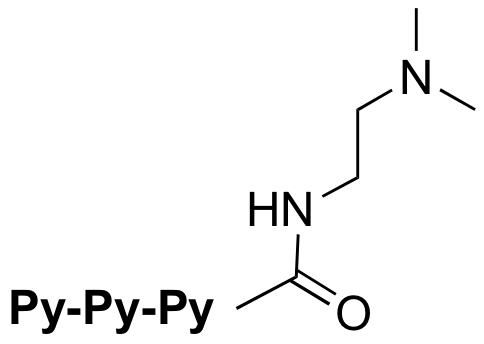 | **1.2** |  | 0.043 | A |  |
| 7 | 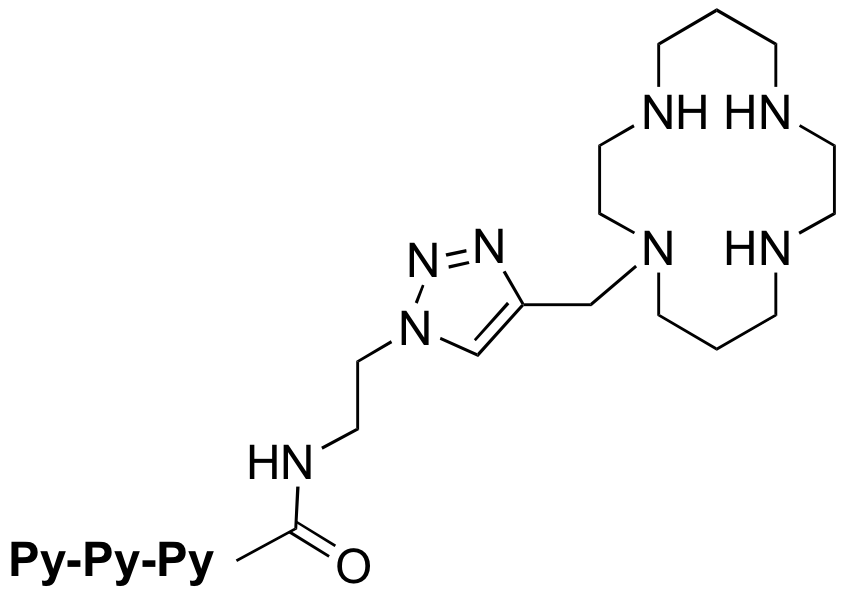 | **2.7**^a^ | CGGCCG | nd | C | - |
